# Fundamental Aspects of Stretchable Mechanochromic Materials: Fabrication and Characterization

**DOI:** 10.3390/ma17163980

**Published:** 2024-08-10

**Authors:** Christina Tang

**Affiliations:** Chemical and Life Science Engineering Department, Virginia Commonwealth University, Richmond, VA 23284, USA; ctang2@vcu.edu

**Keywords:** structural color, polymer, mechanochromic, responsive, dye, strain, elastic, photonic, cellulose nanocrystal, liquid crystal elastomer

## Abstract

Mechanochromic materials provide optical changes in response to mechanical stress and are of interest in a wide range of potential applications such as strain sensing, structural health monitoring, and encryption. Advanced manufacturing such as 3D printing enables the fabrication of complex patterns and geometries. In this work, classes of stretchable mechanochromic materials that provide visual color changes when tension is applied, namely, dyes, polymer dispersed liquid crystals, liquid crystal elastomers, cellulose nanocrystals, photonic nanostructures, hydrogels, and hybrid systems (combinations of other classes) are reviewed. For each class, synthesis and processing, as well as the mechanism of color change are discussed. To enable materials selection across the classes, the mechanochromic sensitivity of the different classes of materials are compared. Photonic systems demonstrate high mechanochromic sensitivity (Δnm/% strain), large dynamic color range, and rapid reversibility. Further, the mechanochromic behavior can be predicted using a simple mechanical model. Photonic systems with a wide range of mechanical properties (elastic modulus) have been achieved. The addition of dyes to photonic systems has broadened the dynamic range, i.e., the strain over which there is an optical change. For applications in which irreversible color change is desired, dye-based systems or liquid crystal elastomer systems can be formulated. While many promising applications have been demonstrated, manufacturing uniform color on a large scale remains a challenge. Standardized characterization methods are needed to translate materials to practical applications. The sustainability of mechanochromic materials is also an important consideration.

## 1. Introduction

Mechanochromic materials that demonstrate dynamic optical properties in response to mechanical stress, e.g., stretching, compression, and bending, are of interest in a broad range of potential applications such as strain sensing, structural health monitoring, and encryption [1,2,3,4,5]. There are two mechanisms of mechanochromism: the chemical conversion of mechanophores (dyes) and physical changes in periodic nanostructure (structural color) [6,7].

These two approaches (chemical or physical) to achieving mechanochromic properties have typically been reviewed separately. For example, mechanochromic dyes have recently been reviewed [8], and structural approaches have been reviewed separately [4,9]. Reviews that have included both chemical and physical approaches have focused on applications, e.g., structural health monitoring [7] and electronic devices [10]. However, these reviews including both chemical and physical approaches determined that it was difficult to compare different mechanochromism strategies [7]. Therefore, the goal of this work is to compare the functional performance of chemical-based and physical-based mechanochromic, elastic systems used in tension.

In this work, mechanochromic materials that provide visual color changes when tension is applied are reviewed. The mechanochromic response to other mechanical stimuli (e.g., compression and bending) have recently been reviewed elsewhere [7] and are outside the scope of this work. Such an affect has been achieved with various classes of materials including mechanochromic dyes, polymer dispersed liquid crystals, liquid crystal elastomers, cellulose nanocrystals, photonic nanostructures, hydrogels, and hybrid systems (combinations of other classes). For each class of mechanochromic material system, synthesis and processing, as well as the mechanism of color change are discussed. Representative examples of each class of material are provided with an emphasis on visual color change.

For quantitative comparison across the different classes of mechanochromic materials, the mechanochromic sensitivity (Δnm/% strain) was evaluated as a function of elastic modulus. Additional properties such as dynamic color range and reversibility are briefly discussed. This comparison is expected to enable materials selection across the various classes of materials for diverse applications of interest. A brief outlook on emerging applications and current gaps in the path to commercialization are provided.

## 2. Dyes

The use of mechanochromic dyes is a well-established approach for achieving optically responsive materials. In mechanochromic dyes (mechanophores), the application of an external force selectively affects the weak bonds in the molecule resulting in bond cleavage or isomerization. The mechanical activation of mechanophores can be accompanied by optical changes such as shifts in absorption (visible color) or emission (fluorescence or luminescence) [11].

Mechanophores showing visible color change mainly involve ring-opening isomerization or radical generation [11]. The most common class of mechanophores involves C-O, C-N, or C-S bond breakage of a bicyclic strained structure to form a conjugated colored species (i.e., isomerization based mechanophores) [11]. For example, spiropyran, rhodamine, and naphthopyran undergo reversible ring opening when a loading force is applied [11]. Spiropyran is colorless [11]. When mechanically activated, it turns purple due to ring-opening isomerization to merocyanine [11]. The change in optical properties can be detected with absorbance (band between 500 and 600 nm) or an increase in fluorescence intensity [11]. Similarly, rhodamine (spirolactam-based compound) undergoes ring opening triggered by external (e.g., mechanical) stimuli [11]. The resulting isomer is pink and fluorescent [11]. The color change can be reversed with thermal or optical stimulation [12]. Reversal can be hampered by kinetic factors which leave the mechanophore in its cleaved state. The changes may also result in a permanent loss of mechanical strength [3].

Radical-based mechanophores undergo a homolytic “C-C” bond cleavage of lactones, substituted nitriles, or hydrocarbons under deformation resulting in radical formation accompanied by a color change. Dimers formed from these highly stable carbon-centered radicals are in thermal equilibrium with their radical precursors. This thermal equilibrium facilitates reversible bond cleavage recombination. Examples include diarylbibenzothiophenonyl (DABBT), and diarylbibenzofuranone (DABBF). The radicals generated from DABBF are blue. DABBT derivatives change from colorless to green upon radical generation [11].

For practical applications, mechanochromophores (dyes) are often incorporated into polymers. Dyes can be dispersed in the polymer solution or melt. This blending approach has been of interest because new properties can be achieved without synthesizing a new polymer structure. With blending, a phase separation of the dye and polymer is likely. Thus, the control of the phase of the dye dispersed within the polymer matrix is a fundamental issue governed by affinity/miscibility between the components. Ideally, supramolecular aggregates of dye can be formed within the polymer matrix, i.e., “aggregachromic” dyes. For this class of dyes, color change occurs due to a dimerization and aggregation of the dye. When a mechanical force is applied to the polymer–dye system, macromolecular chain slippage and reorganization result in a breakup of non-covalent interactions of the dye aggregates within the polymer matrix. The breakup of the dye aggregates occurs under plastic deformation and is thus irreversible. Typically, to monitor polymer stretching, signal intensities of monomeric absorption and/or fluorescence and the contribution from the aggregate formed are used. Specifically, the dyes show UV/vis absorption or fluorescence due to the π-π stacking interactions of the planar aromatic backbones. The dye aggregates show changes in the absorption band compared to the monomer [13].

Dye-based mechanochromic systems can also be achieved by covalently linking mechanochromic dyes to polymers. In the case of polydimethylsiloxane (PDMS), a catalytic functionalization of the polyhydromethylsiloxane is performed to covalently attach the dye molecule to the polymer backbone. Mechanophores can also be integrated into difunctional monomers (e.g., diacids, diamines, or glycols) for incorporation into the polymer backbone of polyesters, polyurethanes, etc. [13]. For example, the ring opening of the spiropyran can be mechanically activated when its two ends are tethered to a polymer backbone. Under uniaxial stress, the conversion of spiropyran to merocyanine results in a color change of the polymer from yellow to purple. The spiropyran (colorless/yellow) color is recovered upon radiation with visible light. To achieve color change, the tensile strength must be applied across the spiro-junction to cleave the C_spiro_-O bond between the indoline and benzopyran moieties. This cleavage can be achieved by tethering the polymer chains to opposite ends of the spiro-junction, e.g., the 7- or 8- position of the benzopyran and the 5′ or 6′ position of the indoline. Functionalizing the spiropyran at other positions prevents force transduction across the reactive bond [14]. The strength of covalent bonds in mechanophores affecting the force required to achieve color change can be varied by the attachment of substituents [3]. The properties of spiropyran derivatives have been reviewed elsewhere [14]. Notably, the threshold stress/strain for the color activation of various spiropyran based materials cannot be directly compared as they are affected by the testing methods and conditions [11].

When incorporating dyes into polymers, the attachment position to the polymer can affect the force required for bond cleavage. For example, different attachment positions of naphthopyran were compared when it was incorporated into PDMS. The force required to achieve color change from colorless to yellow for attachment in the 5- position was significantly lower than the 8- or 9- position. This result was attributed to the lower energy required to break the bond as well as the angle between the applied force and the bond. The color and color fading could be tuned by chemical modifications at the 5- position before crosslinking into PDMS. Such tunable properties may be useful for designing multi-color switching materials. The attachment of rhodamine B in multiple locations has been performed to achieve multiple color switches (i.e., blue to pink to yellow under fluorescence). Chemical modifications of mechanophores (e.g., substituting an electron withdrawing group on the 6- position of spiropyran) can affect the kinetics of the conversion of the forward and reverse reactions of spiropyran to merocyanine. Tuning the kinetics can be used to achieve color fading effects. The effect of chemistry on mechanochromic properties have been recently reviewed elsewhere [11].

Additional polymer properties can also affect functional mechanochromic properties. For example, since stress tends to accumulate in or near the middle of polymer chains, mechanophores are normally centered in the polymer backbone unless multiple mechanophores are incorporated. Polymer chain length is also an important consideration. When repeat units on each side of the mechanochromophores were higher than 30, no bright color was observed due to inefficient force transmission to the mechanophore. Using a mechanophore covalently linked to multiple polymer chains (e.g., linear four-arm or eight-arm) has been considered. Interestingly, the ratio of mechanophores that dissociated increased with increasing arm number. With dendrimers, increasing the number of branches led to a higher ratio of cleavage. This result was attributed to improved force transmission and the suppression of the recombination of radicals with increased branches [11]. Polymer chain mobility is also an important consideration affecting the mechanochromic performance of polymer/dye systems. For example, when spiropyran was located in the hard segment, a higher mechanical activation (i.e., strain required for color change) was observed than when spiropyran was located in the soft segment. For radical-based mechanophores, the polymer chain mobility is also an important consideration. Rigid domains have been introduced to enhance mechanochromic properties. This effect is attributed to dissociation occurring at hard–soft interfaces, and the hard domain content reducing the recombination of the colored radicals [11]. Here, a few illustrative examples of the various classes of mechanochromic dyes (e.g., aggregachromic, ring-opening, and radical-generating) incorporated into various elastic polymers (e.g., polyurethane or PDMS) are presented.

**Figure 1:**
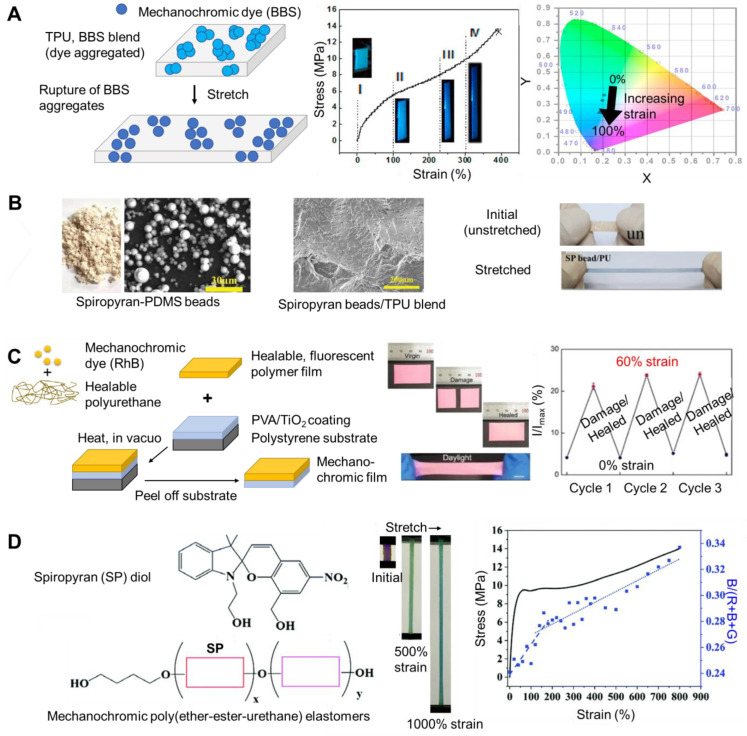
Overview of mechanochromic dyes incorporated into polyurethane elastomer systems. (**A**) Bis(benzoxazolyl)stibene (BBS), an aggrechromic dye, blended with polyurethane. Visible changes were observed under UV light due to disaggregation of the dye upon stretching; changes in hue (images numbered with roman numerals for reference) and brightness were quantified on a chromaticity diagram. Adapted with permission from [15]. (**B**) Bis-alkene functionalized spiropyran was incorporated into PDMS beads and the beads were blended into polyurethane. The resulting films turned from yellow to blue when stretched. Adapted with permission from [17]. (**C**) Rhodamine was blended with polyurethane (self-healable) and layered with PVA/TiO_2_. The mechanochromic response was repeatable after self-healing as quantified by the change in relative fluorescence intensity at 0% and 60% strain. Adapted with permission from [18]. (**D**) Overview of synthesis of mechanochromic poly(ether-ester-urethane) elastomer containing spiropyran. The films turned from yellow to purple with strain. The average intensity of the blue channel B/(R + B + G) was used to monitor the onset of color activation. Adapted with permission from [19].

An example of an aggregachromic dye/polymer system was bis(benzoxazolyl)stilbene (BBS), an aggregachromic dye, blended with a commercial, thermoplastic polyurethane (TPU) and solution cast. In the blend, BBS is aggregated. Upon stretching, changes in the orientation of the TPU induced a rupture of the BBS aggregates which resulted in an increase in fluorescence intensity at 490 nm. Changes were quantified by the ratio of intensity at 490 nm normalized by the intensity at 413 nm. Visible changes upon stretching were observed under a 365 nm UV light (Figure 1A). Changes in hue and brightness were quantified and visualized on a chromaticity diagram (Figure 1A). A visible change in fluorescence was observed at stresses as low as 1 MPa. Full recovery was achieved after annealing at 120 °C (verified by fluorescence intensity) [15]. In a similar system, image analysis has also been used to relate the RGB intensity to the excimer to monomer ratio for BBS/polypropylene blend films that have been strained [16].

Macromolecular dyes such as excimer-forming cyano-substituted oligo(p-phenylenevinylene)s (OPVs) have also been used as mechanophores. When blended with polymers (e.g., polyethylene), the mechanochromic response due to the breakup of the dye aggregates by the polymer host was primarily influenced by the size of the dye aggregates, polymer crystallinity, and strain rate. The size of the dye aggregates was tuned by chemical modification (e.g., a long aliphatic chain introduced to the dye promotes the formation of small aggregates) and dye concentration. Decreasing strain rate increased color change due to the viscoelastic response of the host polymer. The crystal morphology of the host polymer also affected the mechanochromic response [20]. Using confocal microscopy, the deformation of the phase-separated dye inclusions have been visualized during strain [21].

In other approaches, other classes of dyes, such as ring-opening mechanophores, have been blended into polymer matrixes [22]. To achieve mechanochromic systems, bis-alkene functionalized spiropyran was incorporated into beads using emulsion polymerization. The beads were dried then blended with polyurethane (melt processing). The resulting films turned from yellow to blue when stretched (Figure 1B). Fluorescence intensity increased with increasing strain. Thus, fluorescence images were used to visualize nonuniform stress fields. The measured stress distributions agreed with finite element simulations. Alternatively, the beads were blended with polyurethane and solvent or coated on polyurethane. For samples with the same amount of dye-loaded beads, the mechanical properties were comparable and the mechanochromic response was significantly higher for the coated sample than the blended sample (2.5-fold higher fluorescence intensity at 100% strain). This result was attributed to the dye being on the surface of the sample, rather than blended throughout the sample. The coating was patterned and applied to other materials (e.g., aluminum) and used for damage detection [17]. Mechanochromic dyes have also been coated on yarns using dip coating to achieve color changing properties [23,24]. A similar approach has been used to incorporate hydrophobic spiropyran into hydrogel matrices. Dimethylacrylate-functionalized spiropyran (SP) was synthesized (six-step process) and used as a crosslinker when copolymerized with hydrophobic methyl acrylate (MA) monomers and hydrophilic acrylamide (AM) monomers in the presence of surfactant polysorbate 80 micelles. The polymethacrylate/SP was incorporated into the hydrophobic core of the micelles (via hydrophobic interactions), and the resulting micelles were incorporated into the hydrophilic poly(acrylamide) (PAM) network using a one-pot method. The as-prepared spiropyran loaded gel appeared light yellow and transparent to the naked eye, indicating that the spiropyran was well dispersed. Color change from yellow to purple was visible upon strain. The color change was monitored using image analysis using chromaticity (CIE 1931). The color change was reversible with exposure to white light (8 W) for ~10 min [25] or by changes in pH (alkali) [26].

Responsive, self-healing materials have been achieved by blending rhodamine B with polyurethane (self-healable) and layering onto polyvinyl alcohol (PVA)/TiO_2_. Due to the rhodamine B, the fluorescence emission intensity (at 597 nm) increased with increasing strain (quantified with relative intensities). When annealed at 60 °C for at least 3 h after rupture, the mechanical properties were recovered. The self-healing was attributed to hierarchical hydrogen bonding interactions of 2-ureido-4[1H]-pyrimidinone, urethane, and urea. The mechanochromic response was repeatable after self-healing as quantified by the change in relative fluorescence intensity at 0% and 60% strain (Figure 1C). The dye-containing material could be patterned for encryption applications. The approach was versatile; other colors were obtained by incorporating alternative dyes such as fluorescein [18]. Notably, the mechanochromic properties of self-healing polymer blends without dye have also been considered. The change in transmission was quantified with image analysis (chromaticity, blue intensity B/(R + B + G)) [27].

Ring-opening mechanophores can also be incorporated directly into the polymer chain. For example, spiropyran diol has been chemically embedded into poly(ether-ester-urethane) thermoplastic elastomer using poly(butylene terephthalate) (PBT) as the hard segments and poly(tetramethylene glycol) as the soft segments. The resulting polymers could be stretched up to 450% strain (Figure 1D). The modulus decreased with increasing soft segment content. Upon stretching, the films turned from yellow to purple. The onset of color change was ~158%. The change in color was quantified using RGB analysis. The average intensity of the blue channel B/(R + B + G) was used to monitor the onset of color activation. The color change was reversible with exposure to light [19]. As an alternative to polyurethane, spiropyran-diol has been incorporated into polycaprolactone (PCL) via ring-opening polymerization. The resulting polymer films were mechanochromic; the color changed from pale yellow to blue in the stress hardening region (monitored using the blue intensity B/(R + B + G)). Shape memory properties were also observed [28]. Dimethylacrylate spiropyran was also incorporated into poly(acrylamide-co-methyl acrylate) via emulsion polymerization. The resulting polymers were mechanochromic and upon stretching there was a color change from yellow to blue-gray. A linear correlation between tensile strain and chromaticity was observed [29]. Spiropyran diol has also been incorporated into a polyurethane backbone with a tridentate ligand (2,6-bis(1,2,3-triazol-4-yl)pyridine). In the presence of transition metal ions (e.g., Zn^2+^), metallosupramolecular films were formed. The resulting films were mechanochromic turning from brown to blue upon stretching, then red upon fracture. The metallosupramolecular interactions were kinetically labile, which facilitates self-healing properties. The mechanical properties could be recovered after cutting or exposure to solvents [30].

**Figure 2:**
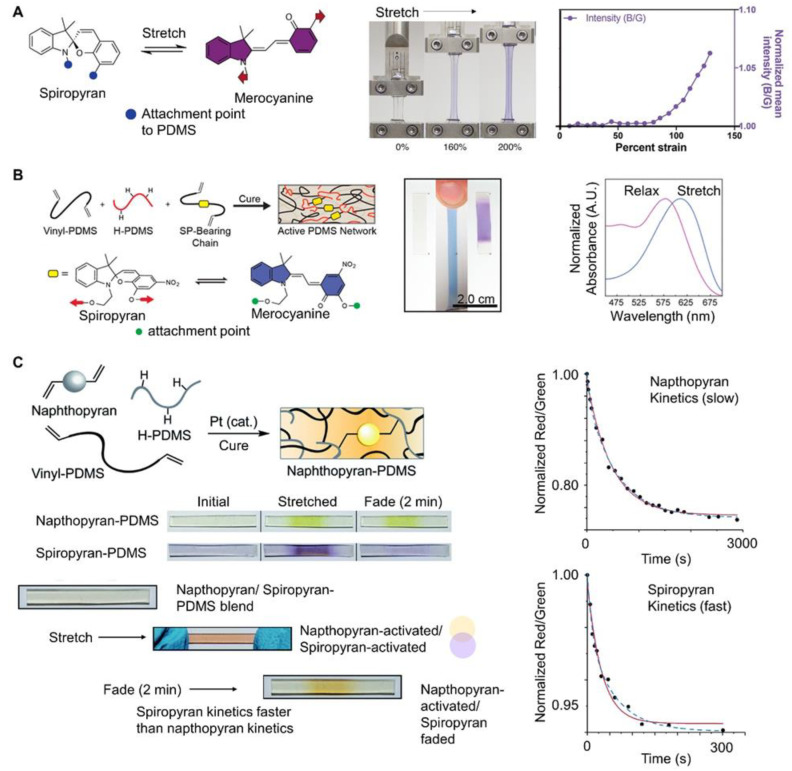
Overview of mechanochromic dyes incorporated into silicone elastomers (PDMS). (**A**) Spiropyran with alkene functionality was added as a crosslinker to commercially available silicone elastomer (e.g., Sylgard 184). Under strain, spiropyran (initially colorless) underwent a ring-opening transition to merocyanine (purple) as a visual indicator of strain. The color change was monitored by changes in the B/G channel intensity. Adapted with permission from [31]. (**B**) Bis-alkene functionalized-spiropyran incorporated into PDMS. The original sample (colorless) turned blue when stretched, and purple when released to its initial shape. Arrows indicate ring opening. The color change was quantified by absorbance spectra. Adapted with permission from [32]. (**C**) Mechanochromic PDMS network incorporated multiple dyes: naphthopyran (orange, slow kinetics) and spiropyran (purple, fast kinetics). Lines (red and blue dashed indicate models fit to the experimental data). Both dyes were activated when the sample was stretched resulting in a pink color. Spiropyran faded faster and after 2 min naphthopyran was activated and spiropyran faded, resulting in a transition from pink to orange. Adapted with permission from [33].

PDMS has also been a widely used elastomer for mechanochromic systems. For example, spiropyran with alkene functionality was added as a crosslinker to commercially available silicone elastomer kits (e.g., Sylgard 184). Due to the spiropyran, the resulting polymer changed color when stretched (i.e., colorless to purple). The color was monitored by image analysis as a visual indicator of strain. Specifically, the changes in B/G intensity were used to quantify changes (Figure 2A). The changes in the slope of the B/G ratio corresponded approximately to the beginning of the strain-hardening region of the stress–strain curve. Mechanoresponsive patterns were achieved by casting mechanochromic PDMS in a mold and partially curing it, followed by casting Sylgard 184 (no dye). Initially the sample was transparent. The pattern appeared upon deformation and the appearance was reversible [31]. Similarly, bis-alkene functionalized-spiropyran was incorporated into PDMS (Sylgard 184) via platinum cure hydrosilylation in xylene. The original solvent cast sample was clear and colorless under ambient conditions, and turned blue when stretched. When released, the material returned to its initial shape, and the color switched from blue to purple. The color change was quantified by absorbance spectra (Figure 2B). The film returned to colorless when exposed to bright white light (10 s) (or 1 h without light). The color change with stretching was repeatable over multiple cycles [32].

Multiple mechanochromic dyes have also been incorporated into the same polymer. The kinetics of the dyes were tuned to achieve multiple color switches. The kinetics of the dyes were evaluated using UV absorbance over time. Each dye was incorporated in PDMS separately and the fading of the color change was monitored using image analysis (R/G compared to the initial) over time (Figure 2C). A PDMS network containing both naphthopyran and spiropyran (covalently bound) was prepared. Naphthopyran (orange, slow kinetics) and spiropyran (purple, fast kinetics) were both activated when the sample was stretched resulting in a pink color. Due to the relatively fast kinetics of spiropyran, it faded faster and 2 min after stretching, naphthopyran was activated and spiropyran had faded, resulting in a transition from pink to orange (Figure 2C) [33]. Incorporating multiple dyes into the same polymer matrix has also increased the dynamic range (i.e., strain over which optical response was observed) of the mechanochromic response since the activation of dye 1 occurred at low stress and the activation of dye 2 occurred at higher strain and overall multiple color changes are observed [34]. Rhodamine has been used with an anthracene-maleimide adduct in a triple polymer network. The anthracene-maleimide was the crosslinker for the first network; the rhodamine was the crosslinker for the second network. At low tensile stress (<1.8 MPa), blue fluorescence was observed due to the activation of the anthracene-maleimide. With further increases in stress, red fluorescence was observed due to the activation of both mechanophores [35]. The incorporation of two radical-based dyes into the hard and soft domains of a blend polymer resulted in material that could discriminate between grinding (pink) and stretching (green) [36].

Typically, mechanochromic/dye polymer systems have been processed into films. Additive manufacturing (e.g., 3D printing) has been used to encase mechanochromic dyes within commercial polymers and may enable a rapid prototyping of multicomponent force sensors [37]. The processing of responsive fibers has also been achieved based on a hybrid phenol–rhodamine dye. The mechanochromic dye was incorporated into a double polymer network. The diacrylate dye was used as a crosslinker for the first polyelectrolyte network of poly(2-acrylamido-2-methyl-1-propanesulfonic acid) (PAMPS). The first network was swollen (this pre-stretching enhanced the stress sensitivity); the second network was formed by photocrosslinking the neutral PAM network. The composite fibers changed from colorless to red when strained (to ~300%) under ambient light or from green to light under UV light. The fibers could also be patterned [38]. The color change was different than typically expected for rhodamine 6G derivative mechanophores, which would shift from red to yellow fluorescent color when stretched [39].

Overall, synthetic routes for mechanochromophores are well established and leverage commonly used approaches in organic synthesis [11]. The force required for color change has been theoretically predicted. Combining experiment and theory, forces on single chains were determined to be a maximum of ~1 nN. Reconciling this force with the macroscopically measured stress of ~50 MPa is an ongoing area of investigation [40]. Dye-based mechanophores typically display two different colors. Systems with multiple color transitions are useful for improving potential sensor and device applications. Multicolor switching systems have been achieved [3]. The resulting change in visible color, absorbance, or light emission can be characterized by RGB color analysis, absorption measurements, or fluorescence imaging [11]. Based on the visible color changes, stress mapping has been demonstrated [41], and identifying areas of high stress after failure at high strain rates (>10^3^ 1/s) [42]. Stress visualization in composite systems using confocal microscopy has also been performed and correlated to calculated stresses from finite element analysis [43]. The methods and conditions (e.g., strain rate) of mechanical testing affect the mechanochromic response [3]. Thus, easy-to-establish and reliable testing methods are necessary for the development of this field [11]. In particular, in situ optical photo capturing is useful for mechanochromophores with visible color changes and is promising for practical use [11]. Such capabilities may enable non-invasive, in situ, stress–strain measurements [44]. Potential applications include force sensing in applications in emerging applications such as the Internet of Things, bioelectronics, and wearable electronics where force sensors with improved flexibility are desired. Common limitations include drift, slow response times, complex fabrication, or limited dynamic measurements. Future work is needed in terms of reproducibility, sensitivity, and response time. The application of these materials in force sensors requires integration within a wider technology system, calibration, and a standardization of testing [45]. While many dye–polymer systems have been considered, multiple synthesis steps are often required which are high in cost and difficult to commercialize [11].

## 3. Polymer Dispersed Liquid Crystals

As an alternative to mechanochromic dyes, liquid crystals have also been used as the basis for responsive (e.g., mechanochromic) materials. Liquid crystals are fluids with long-range order. Nematic phases exhibit only directional order, but no positional order, i.e., all of the molecules tend to orient in one direction referred to as the molecular director, *n*. The nematic phase can be further organized in a cholesteric phase in which planes of molecules exhibit a helical superstructure (cholesteric). The self-assembly of liquid crystals can be directed using a surface alignment layer, shear forces, stretching, and electric or magnetic fields [46]. Cholesteric liquid crystals are promising for structurally colored, mechanochromic materials [4]. When the helical pitch (defined as the unit light of one complete rotation of the director) is comparable to the wavelength of visible light, the samples show structural color due to selective Bragg reflection [47]. The apparent color (wavelength) of reflected light is related to the helical pitch by

$$\lambda =\bar{n}p\cos\theta$$

where *λ* is the wavelength of reflected light, *p* is the pitch length, *θ* is the angle of incident light, and $\bar{n}$ is average index of refraction (~1.6) [48]. The color is iridescent and depends on the angle of incident light; a blue shift is observed when the angle changes from 90°. Thus, the reflected color can be tuned by varying the pitch of the self-assembled liquid crystal molecules. Practically, the pitch is affected by the composition. For mixtures containing chiral dopants, the pitch is determined by

$$p=c^{-1}{HTP}^{-1}$$

where *p* is the pitch, *c* is the concentration of the chiral dopant (molarity or wt.%), and *HTP* is the helical twisting power of the chiral dopant (molarity × µm^−1^ or µm^−1^). The *HTP* of a chiral dopant indicates its efficiency in inducing a twist in a nematic twist. Depending on the liquid crystal components, the pitch (and resulting optical properties) can respond to heat, electric field, or light [46].

Liquid crystal formulations with mechanochromic properties have been reported. Changes in the color and polarization of the reflected light upon uniaxial stretching have been explained by changes in the helical structure (i.e., shorter pitch length due to helix compression) [49]. To achieve macroscopic, responsive color, the mechanochromic cholesteric liquid crystal formulations are generally sandwiched between two parallel planes. Planar anchoring (i.e., helical axis is perpendicular to the plane) is required for a uniform selective Bragg reflection. For stretching, the liquid crystal formulations have been dispersed in polymers. When cholesteric liquid crystals are emulsified into droplets, planar anchoring with the helical axis pointing radially outward is achieved. Thus, a uniform selective Bragg reflection is achieved from the center of each droplet and weak macroscopic color visible to the naked eye is achieved [50,51]. The use of the dispersed cholesteric liquid crystal droplets in elastic polymer films to achieve mechanochromic systems is a promising approach [52,53].

**Figure 3:**
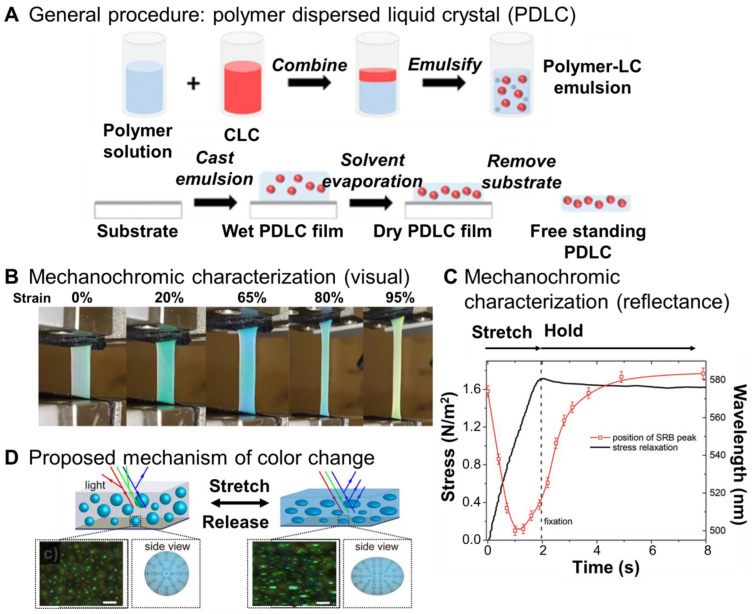
Overview of mechanochromic polymer dispersed liquid crystals. (**A**) Fabrication process in which the liquid crystal (CLC) is mixed and emulsified with a polymer solution and cast into a film. Adapted with permission from [54] (under open access CC-BY). (**B**) Using cholesteryl ester liquid formulations dispersed in polyurethane, films that changed color (e.g., green to blue upon initial stretch) were achieved. Adapted with permission from [55]. (**C**) The mechano-optical response was characterized by UV reflectance as the sample was stretched and then held at 150% strain. A blue shift is observed upon initial stretching followed by a red shift. Adapted from with permission [55]. (**D**) The proposed mechanism of color change is deformation of the liquid crystal droplets into an oblate shape upon mechanical strain. Adapted from [54] (under open access CC-BY) and with permission from [56].

For example, a liquid crystal formulation (E7/S811) was emulsified with polyvinyl alcohol/glycerol (aq.) in a 10/90 ratio via shear mixing. The resulting emulsions were cast on a prepared polyethylene terephthalate (PET) substrate and could be removed after drying (Figure 3A). The responsive properties of the liquid crystal to temperature were retained in the polymer film. Upon uniaxial strain, a deformation of the liquid crystal droplets under polarized light microscopy was observed, indicating a spherical to prolate droplet transition. Increased reflection intensity was observed without a change in color with stretching [54].

Cholesteryl ester mixtures have also been formulated and dispersed in polyurethane (ClearFlex 50, a two-part curable elastomer). The liquid crystal formulation was prepared and combined in component A. The addition of part B resulted in phase separation of the liquid crystal into micron-sized droplets. The mixture was cast and cured in Teflon cells. The resulting films were slightly opaque and colored. The selective reflection band was blue shifted by 30–40 nm when compared to the liquid crystal formulation in a glass cell. This result was attributed to light scattering at the liquid crystal–polymer interface. Upon uniaxially stretching, an initial blue shift in selective reflection band (due to droplet deformation) and increase in reflection in intensity was observed. With further lengthening, a red shift in the selective reflection band was observed. Visually, a sample that was initially pale yellow/green turned blue at 65% engineering strain (blue shift), then back to yellow (red shift) with further increases in strain (Figure 3B). The red shift was confirmed by changes in peak reflectance (Figure 3C) and was attributed to the relaxation of the liquid crystal. The color change kinetics were affected by the stretching rate as well as the viscoelastic response of the liquid crystal [55]. Similar results have been reported with a mechanoresponsive liquid crystal formulation dispersed in PVA/glycerol. The composite was photopatterned, then the pattern could be visualized with mechanical stimuli [57].

Additional color-changing systems have been achieved with other liquid crystal formulations. For example, 5CB/R5011 was emulsified with carbomer/glycerol (Figure 3D). The emulsion could be cast into films or 3D printed. The resulting structures were opaque and white due to the liquid crystal droplets. When uniaxially stretched, a color change from white to blue was observed at ~110% strain. The color change was attributed to the elongation of the liquid crystal along the stretching direction into a prolate shape. The deformation of the droplet affected defects (point to ellipse defects) in the liquid crystal (Figure 3D) affecting the selective refection band (and the apparent color). Under biaxial stretching, the prolate liquid crystal droplet deformed to oblate droplets resulting in the ellipse defect transitioning to a ring defect further affecting the selective reflection band. A color change to orange was observed under biaxial stretching. Responsive shapes (e.g., stars) were achieved by 3D printing [56].

Overall, PDLC systems are mechanochromic systems with unique, tunable dynamic responses. Using cholesteric liquid crystals, mechanochromic materials with multiple color changes can be achieved. Since the liquid crystal/polymer emulsions can be processed via solvent casting and 3D printing, it is a promising approach for achieving functional materials and smart devices.

## 4. Liquid Crystal Elastomers

Liquid crystal elastomers have been used as alternatives to polymer dispersed liquid crystals. In liquid crystal elastomers, the self-assembled structure of liquid crystals with optical properties of interest can be fixed by polymerization (typically photopolymerization) if the liquid crystal monomers contain polymerizable groups. Liquid crystal elastomers can be achieved by ensuring the glass transition temperature of the polymer is below room temperature [46]. The liquid crystal phase of the elastomer is stabilized by intermolecular forces between liquid crystal molecules (mesogens) such as hydrogen bonding and π-π interactions (similar to low-molar-mass liquid crystals). Thus, the phase is responsive to external stimuli and the phase transitions are reversible [58].

The architecture of the polymer network is an important factor. For example, the liquid crystal mesogens can be incorporated into the polymer backbone (main chain type) or attached as a side chain (side chain type). The architecture affects the coupling between liquid crystal order and polymer chain formation. The orientation of the liquid crystal domains is also critical; synthesis with controlled liquid crystal orientation is necessary. To achieve macroscopically oriented liquid crystal elastomers, the liquid crystal elastomer synthesis method must be compatible with alignment techniques (e.g., mechanical alignment and surface alignment). Traditional chemistries include hydrosilylation between silicon hydride and alkene, the polyaddition of epoxy and carboxylic acid, or the free radical polymerization of acrylate. Free radical polymerization is advantageous because the alignment of the liquid crystal is decoupled from the polymerization. Low molar mass reactive mesogens are oriented and the resulting liquid crystal orientation can be locked by the polymerization of acrylate groups. Typically, the network density has a high cross-link density and high glass transition temperatures. To reduce the glass transition temperature, the copolymerization of diacrylate and monoacrylate reactive mesogens is often performed. Liquid crystal elastomers achieved using click chemistry have also been explored, including aza-Michael addition, thiol-ene or thiol-yne reactions, and thiol-epoxy reactions. In the aza-Michael addition reaction, a nucleophile such as an amine is conjugated to an electron-deficient alkene molecule such as an acrylate. The reaction can be carried out under mild reaction conditions with high conversions. The unique radical step-growth process results in networks with low shrinkage upon polymerization and high homogeneity. The reaction time can be tailored using different catalysts. The aza-Michael addition reaction can be combined with free radical polymerization in a one-pot reaction. Typically, excess diacrylate liquid crystal monomer is reacted with an amine to form an acrylate-terminated liquid crystal oligomer. The resulting oligomer has a wide nematic window and is amenable to surface alignment. The free radical polymerization of the residual acrylate groups is photoinitiated to fix the liquid crystal alignment. This approach decouples the alignment and polymerization process. Further, the network structure (e.g., the molecular weight between crosslinks), can be tuned by changing the molar ratio of the monomer(s) and the amine or by modifying the chemical structure of the chain extender. Since the method is relatively simple, it has been used widely for the preparation of liquid crystal elastomers. Further details on liquid elastomer chemistries are available elsewhere [58].

When using surface alignment for the orientation of the liquid crystal mesogens, only thin films can be prepared. Using shear alignment, simultaneous deposition and liquid crystal orientation can be achieved with direct ink writing. Following alignment using any approach, the orientation can be fixed via a photoinitiated free radical polymerization of the acrylate group. Thus, liquid crystal elastomers with complex geometries have been achieved by advanced manufacturing techniques, e.g., stereolithography, digital light processing, fused filament fabrication, direct ink writing, etc. For fused filament processing, cholesteric liquid crystal formulations have been adapted into particles and compounded with appropriate polymers. Alternatively, liquid crystal oligomers can be formulated for direct ink writing. Structurally colored, responsive materials that can be 3D printed have been achieved. Processed materials that change color with strain have been achieved with commercially available reactive mesogens and a custom synthesized chiral dopant [59]. An overview of mechanochromic liquid crystal elastomers with responsive optical properties in tension with representative examples is provided.

**Figure 4:**
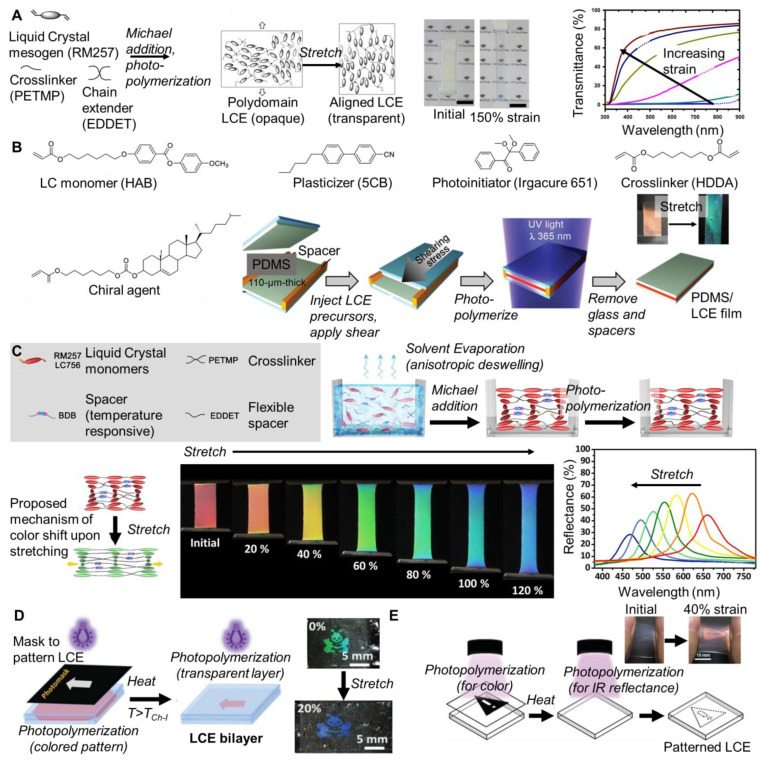
Overview of mechanochromic liquid crystal elastomers. (**A**) Opaque liquid crystal elastomer due to scattering of the liquid crystal domains. Upon stretching, the liquid crystal domains aligned resulting in increased transparency (adapted with permission from [60]). Dotted portions are interpolated based on the plot. (**B**) Structurally colored liquid crystal elastomers obtained by injecting the components: liquid crystal monomer, chiral agent, crosslinker, plasticizer, and photoinitiator into a glass cell lined with PDMS via capillary force, applying shear force for orientation of the cholesteric phase, followed by photopolymerization. The resulting PDMS/liquid crystal elastomer film changes color when stretched (adapted with permission from [61]). (**C**) Structurally colored liquid crystal elastomer films prepared by anisotropic deswelling in which the components are mixed in a solvent and cast on a glass substrate. A film that initially appears red turns blue upon stretching, confirmed with changes in the reflective spectra (adapted with permission from [62]). (**D**) Liquid crystal elastomers prepared on PVA-coated substrates with photomask and multiple crosslinking steps to achieve colored patterns and transparent portions (adapted with permission from [63]). (**E**) Using multiple crosslinking steps and a photomask, the colorless and transparent films could be achieved. Color appeared in the specified pattern upon stretching (adapted with permission from [64]).

For example, the polymerization of a liquid crystal mesogen (RM257) with 2,2′-(ethylenedioxy) diethanethiol (EDDET) and pentaerythritol tetrakis (3-mercaptopropionate) (PETMP) via Michael addition reaction and photopolymerization results in an opaque liquid crystal elastomer. An overview of an example chemistry used to achieve such properties is included in Figure 4A. To achieve a liquid crystal elastomer with responsive optical properties, the network was synthesized at ambient temperature, below the clearing temperature so the domain size of the liquid crystal domain (not aligned) was several hundreds of nanometers. Upon stretching, the liquid crystal domains aligned, and the liquid elastomer became transparent. Notably, the transition from opaque to transparent occurs at relatively large strain (~150%) [60].

Structurally colored, responsive liquid crystal elastomers can be achieved by including a chiral agent to achieve cholesteric liquid crystal elastomers. For example, colored films were fabricated by combining a liquid crystal monomer, chiral agent, crosslinker, plasticizer, and photoiniator (Igragure 651) in appropriate proportions, injecting the mixture into a glass cell lined with PDMS films via capillary force, and applying shear force to obtain a homogeneous orientation of the cholesteric phase, followed by photopolymerization (chemical structures and process overview in Figure 4B). The resulting PDMS/liquid crystal elastomer film was structurally colored and mechanochromic, i.e., it changed color (orange to green) when stretched. The recovery time was significantly faster than the liquid crystal elastomer alone. This result was attributed to the effect of the PDMS layers on the relaxation time of the liquid crystal elastomer layer using the Voigt model [61]. A PDMS layer has also facilitated strain-selective encryption. Under optical microscopy, a red to blue transition was observed when the strain was increased from 0 to 39% strain [65]. The mechanochromic sensitivity of such systems can be low and the color change can be difficult to perceive with the naked eye. For example, in some liquid crystal elastomer systems the color change was not visible until the tensile strain is 100% [66].

Responsive, structurally colored liquid crystal elastomers have also been achieved by two-stage thiol-acrylate Michael addition and photopolymerization. In the first stage, a thiol-acrylate Michael addition reaction was performed in a glass cell. The resulting partially crosslinked film was uniaxially stretched to deform the helical structure while the liquid crystal mesogens aligned along the stretching direction. The film was crosslinked in the stretched state using photopolymerization. The deformed helix reflected both left- and right-handed circularly polarized light (hyperreflectivity). The shape and structural color underwent reversible changes with temperature. For example, upon heating, the film showed uniaxial actuation and decreased in length along the stretching direction upon heating from 22 °C to 171 °C with a change in color from green to red (500 to 680 nm). Dynamic covalent bonds have been incorporated to facilitate reconfiguration. For example, by incorporating allyl dithiol in the backbone of the cholesteric liquid crystal elastomer network, the shape and structural color could be reconfigured when stretched and then fixed by exposing to UV light. The degree of reconfiguration was adjusted by tuning the UV exposure time (bond exchange reaction time) [46].

An alternative approach to liquid crystal elastomer preparation has been anisotropic deswelling in which the components were mixed in a solvent and cast on a glass substrate. The first stage Michael addition reaction proceeded while the liquid crystal molecules self-assembled into helical nanostructures that reflected visible light due to solvent evaporation and an anisotropic deswelling of the film plane. Once the solvent was fully evaporated, the film was photopolymerized. The resulting films showed dynamic mechanochromic properties at room temperature. For example, a film that initially appeared red turned blue upon stretching. The color change was quantified with reflectance spectra (Figure 4C). The selective refection band shifted from 659 nm to 468 nm when the applied strain increased from 0% to 120%. A dynamic bond (boronic ester) was incorporated. Thermo-activated B-O bond exchange programmed their color and 3D shape. Water-assisted B-O exchange facilitated self-healing at room temperature. Thus, these are promising materials for bioinspired camouflage and somatosensory soft robotics [62]. Notably, the substrate used for preparation (e.g., glass compared to PDMS) can affect the initial color. A blue shift was observed in the peak position of the selective reflection band for films prepared on PDMS compared to glass using anisotropic deswelling. This result was attributed to the compression of the helix in the relaxed state prepared on PDMS compared to the helix made on glass [49]. Using anisotropic deswelling, liquid crystal elastomers have been prepared on silane-functionalized polymer ionic liquid networks to achieve mechanochromic, conductive materials for sensor applications [67].

Building on these techniques, patterning liquid crystal elastomers has facilitated more advanced designs. For example, liquid crystal elastomers prepared on PVA-coated substrates were patterned using photomasks. Following patterning and shear alignment with a treated glass slide, a two-stage photopolymerization of the liquid crystal elastomer was performed at 50 °C; the temperature was increased to 65 °C for the photopolymerization of the transparent/invisible parts of the pattern (Figure 4D). A PDMS layer was laminated to the resulting pattern using UV glue. Designs such as an arrow, bee, and octopus were demonstrated. The patterns were responsive to stimuli such as stretch [63]. A similar approach has been used for reversible image reveal. Liquid crystal elastomer was patterned using photomasks and then crosslinked in multiple steps at different temperatures. After coating, the sample was cured at 43 °C through a mask (desired image) (visible/IR). The mask was removed, the sample was heated to 51 °C, and the sample was cured (transparent/IR). The resulting coating reflected in the IR range and the pattern was invisible and the film appeared colorless and transparent. Upon stretching, the reflection band of the desired image portion blue-shifted to the visible range so that the pattern appeared. A proof of concept was demonstrated with a warning sign [64]. Patterns of multiple colors have also been obtained by performing multiple crosslinking steps in the stretched (with photomask for pattern) and unstretched state. Patterns were revealed upon stretching [68]. Photopatterning and crosslinking using different intensities have also been used to achieve multicolor patterns [69].

For patterning liquid crystal elastomers, direct ink writing onto PVA–coated glass has also been used. The resulting mechanochromic properties are anisotropic with respect to the printing direction. Specifically, the mechanochromic response was more sensitive when the strain was perpendicular to the printing direction (1.33 nm/% strain perpendicular compared to 0.56 nm/% strain parallel to the printing direction when observed at normal incidence). This difference was attributed to the tilt of the helix during printing. Overall, printing facilitated the patterning and tuning of the structural color [1]. Direct ink writing onto elastomeric surfaces has also been reported. Logos and chameleons were achieved on black PDMS (Sylgard 184) substrates functionalized with 3-(trimethoxysilyl)propyl methacrylate [70].

**Figure 5:**
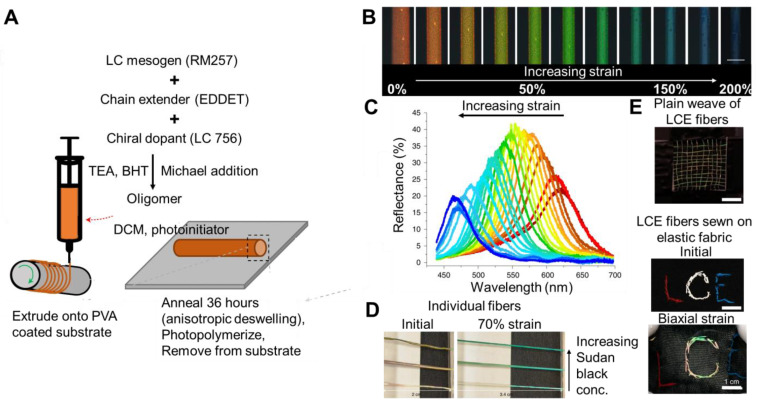
(**A**) Overview of liquid crystal elastomer fibers prepared by anisotropic deswelling via extrusion onto a PVA-coated mandrel. (**B**) Images of mechanochromic response of resulting fibers under increasing strain observed under reflection mode polarized optical microscopy (200 µm scale bar). (**C**) Reflectance spectra of the selectively reflected light obtained through a right-handed circular polarizer. (**D**) Liquid crystal fiber (red) with increasing concentrations of Sudan black against white paper and black cloth under ambient light. (**E**) Resulting fibers woven (pain weave or hand sewn) into black elastic fabric under ambient light with 1 cm scale bars. Adapted with permission from [73].

Liquid crystal elastomers can also be made directly into fibers for textile applications. For example, Lagerwall and co-workers synthesized liquid crystalline oligomer based on a thiol-acrylate Michael addition of liquid crystal mesogen RM257, 2,2′-(ethylenedioxy) diethanethiol (EDDET), and chiral dopant LC756 using triethylamine as a catalyst to initiate the click reaction (Figure 5A). The resulting oligomer (with acrylate end groups) was diluted with dichloromethane and a photoinitiator in an appropriate concentration to facilitate filament formation. The filament was extruded onto a PVA-coated substrate mandrel. The color resulted from anisotropic deswelling as the solvent evaporated. The color of the fiber was tuned by varying the amount of chiral dopant (LC756) used in the process. The resulting fiber was photopolymerized, and the liquid crystal elastomer was crosslinked after the helix was developed. Due to extensional flow during extrusion, the oligomer aligned uniaxially with the direction of long-range orientational order along the filament (from observations of birefringence). Under reflected polarized optical microscopy, the fibers were approximately 250 µm in diameter and were initially red (enhanced with right-handed circular polarization). When elongated, the red transitions through the full visible spectrum (i.e., red to orange to yellow to green to blue) (Figure 5B). The mechanochromic response was immediate and fully reversible. The color change was quantified by measuring the reflectance spectra as a function of tensile strain extension along the long axis of the fiber. Experimentally, the selective reflection band shifted from approximately 615 nm to 520 nm as the strain increased from 0% to 200% Figure 5C. This reflected wavelength has been predicted theoretically using standard mechanical analysis relating the strain to the change in thickness using Poisson’s ratio

$$\lambda =\lambda_{0}{\left(1+\varepsilon_{zz}\right)}^{-\upsilon }$$

where *ε_zz_* is strain, *λ*_0_ is the initial color, and *ν* is Poisson’s ratio. For a typical elastomer, Poisson’s ratio is ~0.5 [71]. This approach has been applied to liquid crystal elastomers in various geometries including films and fibers [71,72].

The mechanochromic response was robust; minimal change was observed after 100 cycles at 200% strain, 10 full laundry cycles, followed by another 100 cycles at 200% strain. Under ambient light, the color change of the fibers could also be seen upon stretching (Figure 5D). The resulting fibers could be woven or sewn into existing textiles to achieve mechanochromic textiles (Figure 5E) [73]. In another approach, fibers were achieved by injecting the oligomer/dichloromethane/photocrosslinker in a tubular template (LDPE) low density polyethylene. Following photopolymerization, the LDPE tube was removed with toluene, and the resulting fiber was dried in vacuo. The resulting fibers were mechanochromic, undergoing a maximum blue shift of 223 nm (726 nm to 503 nm) as the fibers were strained from 0 to 180%. The fibers were used to visualize localized strains in knotted fibers [48].

Overall, liquid crystal elastomers are promising systems for achieving responsive optical properties. Click chemistries have been developed for a relatively fast and efficient synthesis of liquid crystal elastomers that can be decoupled with the alignment of the liquid crystals. Changes in transmission or changes in color with strain have been achieved depending on the liquid crystal formulation. The change in color with response to strain can be predicted theoretically using standard mechanical analysis. The kinetics of the color change reversibility have been tuned by layering the liquid crystal elastomer with other elastic substrates (e.g., PDMS). Patterning (photomasks) and processing using additive manufacturing have been demonstrated to facilitate advanced, responsive designs. Processing liquid crystal elastomers directly into fibers has also been demonstrated, showing the versatility of these materials. With the various chemistries and processing, alignment strategies to achieve the desired properties need to be considered [58,74].

## 5. Cellulose Nanocrystals

Cellulose nanocrystals can self-assemble into nematic structures with responsive structural color [75,76]. Specifically, cellulose nanocrystals (extracted from plants) are crystalline nanorods with a high aspect ratio. In aqueous solution, they self-assemble into a lyotropic cholesteric liquid crystal phase with a left-handed helical twist due to the chiral 1,4-D-glucose units. Films of cellulose nanocrystals are brittle. The cellulose nanocrystals can be integrated with hydrogel, polyethylene glycol, or elastomers to make flexible films. In an elastomer film, responsive optical properties can be achieved with stretching. To achieve responsive, structural color, various hydrophilic small molecules can be co-assembled with the cellulose nanocrystals [46,75,77].

**Figure 6:**
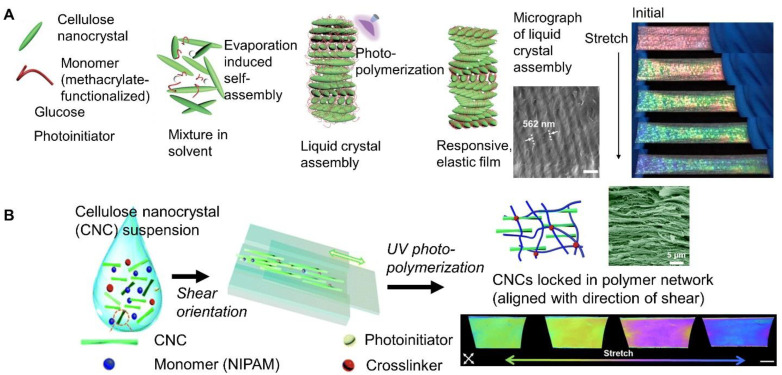
Overview of mechanochromic materials using cellulose nanocrystals. (**A**) Self-assembly of photonic cellulose nanocrystals with glucose and polymer precursors followed by photopolymerization. SEM images of the films demonstrating the layer spacing on the order of visible light. Adapted with permission from [78]. Representative mechanochromic response of cellulose nanocrystal/glucose/polyacrylate films. Adapted with permission from [75]. (**B**) Shear alignment of cellulose nanocrystals dispersed in monomers followed by photopolymerization and swelling to achieving structurally colored mechanochromic films that changed when stretched (viewed under crossed polarizers with a 1 cm scale bar). Adapted with permission from [84].

For example, responsive, structurally colored films were achieved when 2-hydroxyethyl acrylate (HEA) and glucose (Glu) were co-assembled with cellulose nanocrystals, followed by the photopolymerization of the composite films (Figure 6A). The structural color of the film was tuned by varying the glucose concentration, which increased the layer spacing of the chiral nematic structure. By varying the monomer amount, the fracture strain could be increased to 80%. The color change between red and blue was achieved below 62% strain and was reversible. The reversibility was confirmed by reflectance measurements; λ_max_ shifted from 671 nm to 486 nm at 62% strain and returned to 664 nm immediately [75]. A similar approach has been used to co-assemble cellulose nanocrystals and poly(ethylene glycol) dimethacrylate PEGDMA monomers. Structurally colored composites were obtained following free radical photopolymerization. The color of the composite film (purple to red) was tuned by varying the monomer concentration. When stretched, a composite that was initially red changed to yellow (strain ≈ 50%), and to green (strain ≈ 80%). The color change was quantified with chromaticity and attributed to a change in pitch length [78]. The initial wavelength could be tuned to the NIR range resulting in transparent films that appeared red with strain [79]. Using this approach and glycerol as an additive for co-assembly, the composites were prepared in a black PDMS mold. The resulting device was used as a wearable sensor for visually assessing muscle movements during training and correcting posture [80]. Using ionic liquid as the matrix, mechanochromic and conductive materials for sensors were achieved [81]. Incorporating carbon nanotubes with the cellulose nanocrystals and polymer (poly(acrylamide-co-acrylic acid)) enhanced the saturation of the structural color as well as introduced conductivity. When applied as sensors, electrical resistance was used to quantify the stimulus, and optical signals were used to map the mechanical stimulations (e.g., tension) [82]. Introducing gradients during evaporation resulted in gradients of helical pitches. Thus, when the composite was patterned, the resulting film showed no visible color in the relaxed state and the pattern was revealed when the material was stretched [83].

Cellulose nanocrystals have also been shear aligned. For example, cellulose nanocrystals were suspended with N-Isopropylacrylamide (NIPAM) monomers and sheared to align the cellulose nanocrystals. In situ UV photopolymerization was performed to lock in the shear-induced orientation (Figure 6B). Structural color (orange) was achieved when the composite was swollen. When viewed under crossed polarizers, a color change from green to bright yellow to pink during the stretching was observed (Figure 6B) [84]. For cellulose nanocrystals, models of structural color are typically based on mesogen orientation quantified by the order parameter (measured by X-ray scattering). To understand the effect of anisotropic deswelling or stretch, the compression matrix formalism has been used [85,86].

Overall, the self-assembly of cellulose nanocrystals provides a highly tunable platform to achieve responsive structural color including mechanochromic properties. Models to predict the structural color based on the order parameter (measured using X-ray scattering) have been developed [85,86]. The use of materials from renewable sources (e.g., cellulose nanocrystals) is important in addressing the sustainability of mechanochromic materials [59,87].

## 6. Photonic Nanomaterials

Structural colors can also be generated by the physical interaction of light and periodic micro/nanostructures and are found widely in nature, e.g., peacock feathers and butterfly wings. The advantages of structural color include long-term stability. This physical approach is also considered environmentally friendly compared with conventional pigments and dyes (chemicals to achieve the selective absorption of visible light). To tune the color of photonic crystals, the reflected wavelength (i.e., structural color) can be predicted by Bragg’s law as

$$\lambda =2D{\left(n_{eff}^{2}-{cos}^{2}\theta \right)}^{1/2}$$

where *λ* is the wavelength of the reflected light, *n_eff_* is the average refractive index of the components within the photonic crystal, *D* is the lattice constant, and *θ* is the angle of incident light [88]. Such structurally colored materials have been engineered using colloidal assembly, lithography, and top-down engineering techniques [89].

Dynamically responsive structural colors are also possible. For example, in nature, the chameleon [90] and octopus [91] can dynamically and reversibly adjust their surface colors as camouflage based on their surroundings. These characteristics have been, in part, attributed to adaptive structural color [90,91]. In particular, cephalopods can change their appearance in part by stretching or deforming their skin [92], and thus have served as inspiration for synthetic systems, e.g., [93]. Various nanostructures have been used to generate such responsive, structural color including photonic crystals, multilayer films, and metasurfaces [4]. For multilayer films, structural color is based on thin film interference [4]. The reflectivity can be calculated from the transmission matrix theory (described elsewhere [94]) depending on the number of layers, the refractive index of each layer, the thickness of each layer, and the wavelength of incident light, as well as the angle of incidence and polarization of light [4]. Reflected color has also been achieved from metasurfaces with periodic structures achieved by lithography and imprinting processes [4]. The use of flexible materials such as PDMS with photonic crystals has given rise to materials that change color upon stretching [94]. The use of an elastic component is necessary for achieving reversible mechanochromic properties [4].

**Figure 7:**
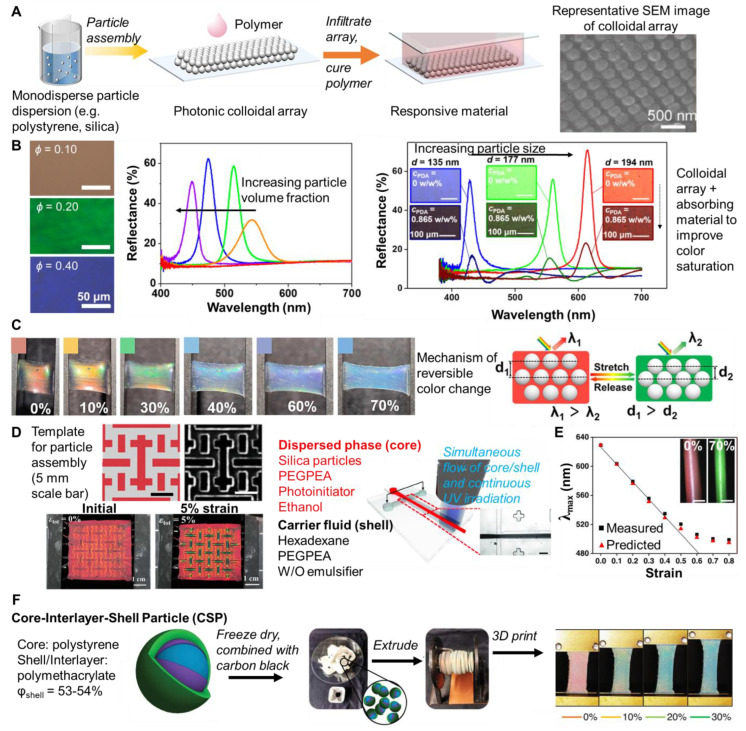
Overview of photonic mechanochromic materials based on colloidal particle assemblies. (**A**) Deposition of monodisperse particles (silica or polystyrene) results in self-assembly of the particles into a colloidal array (ordered hexagonal arrangement). The colloidal array is infiltrated with monomers followed by polymerization, resulting in structurally colored, responsive composites (adapted with permission from [103]). Representative SEM of the particle packing adapted from [104] with permission. (**B**) Effect of particle volume fraction on resulting structural color for 150 nm (diameter) silica particles dispersed in PEGPEA (adapted with permission from [96]). The effect of particle size on resulting structural color for silica particles dispersed in PEGPEA. The use of polydopamine particles (PDA) increased brightness by absorbing diffusely scattered light (adapted with permission from [95]). (**C**) Dynamic mechanochromic response of photonic composite materials. The color changed due to a reduction in lattice spacing upon stretching. Adapted with permission from [103]. (**D**) Colloidal assemblies patterned using custom designed templates demonstrating high mechanochromic sensitivity due to stress concentration in the patterned areas. Adapted with permission from [105]. (**E**) Structurally colored, mechanochromic fibers produced from monodisperse silica using microfluidics. The fibers turned from red to green when stretched. Adapted with permission from [106]. (**F**) Three-layer particles (polystyrene core and polymethacryate interlayer/shell) formulated for 3D printing. An initially red 3D printed dog bone turned from red to green upon increasing the strain from 0 to 30%. Adapted with permission from [98].

A common approach to responsive, structurally colored materials has been colloidal crystals. Mechanochromic systems are achieved by selecting an elastic matrix for the colloidal crystal. Fabrication involves the assembly of the colloidal particles and introduction of the polymer matrix (Figure 7A). In this approach, the periodic photonic structure is first generated by assembling monodispersed colloidal particles via evaporative self-assembly. Subsequently, polymers or monomers are infused into the void spaces and solidified (or the monomers are polymerized). However, capillary force can limit the polymer in filling the voids. Alternatively, the polymer can be introduced simultaneously with particle assembly. Practically, the interactions between the colloidal particles and polymers or monomers (good compatibility) as well as solvent evaporation are important considerations to achieve the assembly of the photonic crystal [89].

The periodic structure of the colloidal particles (the ordering of the colloidal particles and interparticle distance) as well as the refractive index of the colloidal particles and matrix dictate the optical properties, e.g., the structural color. Colloidal particle assemblies with long-range order result in iridescent color (i.e., angle-dependent structural color). In contrast, amorphous, short-range ordered particle arrangements exhibit angle-independent structural color. The interparticle particle spacing also affects the apparent color [89]. Specifically, considering the size and refractive index of the particles, the apparent color (reflected wavelength) of colloidal photonic-based materials is typically predicted as

$$\lambda ={\left(\frac{\pi }{3\sqrt{2}\phi }\right)}^{\frac{1}{3}}{\left(\frac{8}{3}\right)}^{\frac{1}{2}}d_{particle}{\left(n_{particle}^{2}\phi +n_{matrix}^{2}(1-\phi )\right)}^{\frac{1}{2}}$$

assuming a non-close packed face center cubic, where *λ* is the reflected wavelength *ϕ* is the volume fraction of particles, *d_particle_* is the particle diameter, *n_particle_* is the refractive index of the particle, and *n_matrix_*, is the refractive index of the matrix [95]. Thus, the size and the concentration of the particles and the material selection of the particle and matrix are important practical considerations affecting the optical properties of the material (Figure 6B). Notably, the particle volume fraction affects the color [96] as well as the mechanical properties of the composite photonic materials. For silica in polyethylene glycol dimethacrylate (PEGDMA), it has been found that the Young’s modulus, fracture strain, and toughness increased as the amount of silica increased. The changes in Young’s modulus were consistent with the Goth-Gold equation, a classical expression for the elastic modulus for reinforced elastomers for spherical rigid particle suspensions

$$E^{*}=\left(1+2.5\phi +14.1\phi^{2}\right)E$$

where E* is the Young’s modulus of the composite, *E* is the Young’s modulus of the unfilled matrix material, and *ϕ* is the volume fraction of particles. Experimentally, the Young’s modulus of the composite can be higher than theoretically predicted. This deviation has been attributed to the formation of aggregates [97].

Materials selection of the colloidal particle and matrix material also affect the optical properties. Specifically, the refractive index contrast between colloidal particles and matrix impacts the optical transmittance and color visibility. Commonly used polymers for the polymer matrix have a refractive index (*n* ~ 1.40 and 1.55). Particles can be organic, inorganic, or an organic–inorganic hybrid. A uniform particle size, shape, and surface charge of the colloidal particles are required to achieve ordered assembles rather than particle aggregation due to van der Waals forces. Common inorganic particles include silica (SiO_2_, *n* ~ 1.45). Such particles are typically synthesized via the sol gel method, i.e., the hydrolysis of silicon aloxide in a mixture of water and alcohol in the presence of ammonia catalysis. Since the isoelectric point of silica particles is pH 2, neutral or alkaline conditions are appropriate for self-assembly into ordered photonic structures. To increase the refractive index contrast between the polymer matrix and colloidal particles, metal or metallic oxide particles have also considered (e.g., ceric dioxide, *n* ~ 2.20, and cadmium sulfide, *n* ~ 2.52). Iron oxide particles (Fe_3_O_4_-based particles, *n* > 1.70) are also of interest. Alternatively, hollow particles with a low index of refraction have also been examined, e.g., hollow silica particles with *n* ~ 1.10–1.20. Organic (polymeric) particles are also commonly used (e.g., polystyrene (PS) particles with *n* ~ 1.59 and poly(methyl methacrylate) PMMA particles with *n* ~1.50 produced via emulsion polymerization) [89]. To achieve organic particles with a high refractive index, components containing aromatic rings, sulfur elements, and halogen elements (except fluorine) can be used (e.g., polysulfide and polydopamine). The advantages of organic particles include multifunctional groups, low density, and easy modification. The advantages of inorganic particles include a high index of refraction and high mechanical stability. Hybrid organic–inorganic particles, e.g., PS particles coated with silica, have also been considered. The presence of the polymer coating has improved color saturation by absorbing incoherent light scattering and suppressed the coffee ring effect during self-assembly due to increased surface roughness [89]. For core-shell particles, the color has been predicted using the equation

$$\lambda =2{\left(\frac{2}{3}\right)}^{\frac{1}{2}}d{\left(n_{eff}^{2}-{cos}^{2}\theta \right)}^{1/2}$$

where *λ* is the reflected wavelength, *d* is the particle diameter, *n_eff_* is the effective refractive index of the particle, and *θ* is the angle of incident light [98].

Building on these approaches in particle self-assembly, inverse opal structures have also been fabricated using close packed nanoparticles (e.g., silica as templates), and then removing the templates to create highly ordered voids. The reflected wavelength of the inverse opals can be determined by

$$\lambda =\frac{2d}{m}{\left(n_{eff}^{2}-{sin}^{2}\theta \right)}^{1/2}$$

where *λ* is the reflected wavelength, *d* is the distance between particle planes, *m* is the order of diffraction, *θ* is the angle of incident light, and *n_eff_* is the mean effective refractive index [46].

The physical properties of the matrix are also important considerations as the polymeric matrix supports the photonic crystals and provides the composite materials with deformability, desired wettability, etc. Low refractive index contrast between the polymer and the colloidal particles results in insignificant structural color. Thus, light absorbing additives to reduce scattered light have been examined to improve the color visibility [99]. To impart advanced functionalities, self-healing polymers, shape memory polymers, and responsive polymers (e.g., temperature sensitive or light sensitive) have been used as matrixes. The polymer matrix can also provide smart or responsive functions by changing physical properties. Further details on such advanced responsive smart materials combining photonic crystals with responsive matrixes are provided elsewhere [89]. Systems that respond to multiple modes of mechanical deformation are possible, e.g., pressure [100]. Our focus is on mechanochromic systems with visual color change under tension. An overview of recent, demonstrative examples of mechanochromic materials based on colloidal assemblies are discussed below.

For the preparation of photonic mechanochromic materials based on colloidal particles, typically dispersions of monodisperse particles (silica or polystyrene) are prepared and self-assembled via capillary force and solvent evaporation The colloidal array (ordered hexagonal arrangement) is shown in Figure 7A, representative scanning electron microscopy (SEM) To achieve responsive structural color, the colloidal array, infiltrated with monomer followed by polymerization. The shear-based processing of the colloidal dispersions/polymer solutions has enabled continuous (roll-to-roll), scalable processing [101]. The structural color arises from the size and spacing of the colloidal particles. The particle spacing can be affected by particle concentration, i.e., particle volume fraction. With increasing particle volume fraction (for 150 nm diameter silica particles dispersed in PEGPEA) a shift from red to blue was observed (Figure 7B) [96]. Since the structural color is affected by the spacing between layers of particles, the color is also affected by particle size. As particle size decreases, the wavelength of reflected light decreases. For example, at a constant particle volume fraction of 0.33, the color of the film (silica/PEGPEA) was changed from blue to red by varying the particle size from 135 nm to 194 nm in diameter (Figure 7B). The color brightness of the films was also affected by including a light-absorbing material such as polydopamine particles (PDA particles). The PDA particles were added in low concentrations and incorporated in the interstitial regions of the colloidal array. The presence of PDA particles prevented the colloidal crystallization of the silica particles and the long-range order of the silica particles was partially lost. Due to the reduction in long-range order, the films containing PDA particles showed a much lower peak reflectivity and the color brightness was reduced under optical microscopy (Figure 7B). Notably, films containing PDA particles showed higher color saturation under ambient light condition because the PDA particles absorb diffusive scattering light and reduce reflection from the background [95]. Alternatively, iron oxide-based particles have been considered due to their high contrast [102].

Mechanochromic composite materials have also been achieved using the self-assembly of other nanostructures (e.g., Dion-Jacobson type layered perovskite oxide (KCa_2_Nb_3_O_10_) sheets). Aqueous dispersions of single layer sheets of Ca_2_Nb_3_O_10_ were prepared from KCa_2_Nb_3_O_10_. To form composites, a monomer (NIPAM), crosslinker, and photoinitiator were added, and the mixture was cast between silicone and glass. The composite was achieved via photoinitiated radical polymerization, and followed by swelling in water. The material could be patterned using a silicone stamp. The structural color of the resulting nanosheet/polymer composite film was attributed to the swollen lamellar structure. The color could be tuned by varying the perovskite oxide concentration. For a film that was initially orange, the structural color changed from orange to green at 20% strain, blue at 75% strain, and purple at 80% strain. Upon release, the color returned to orange with a response time of less than 1 millisecond [107].

To achieve mechanochromic properties, an elastomeric matrix is commonly used. For example, the multiple hydrogen bonding networks of polyacrylic acid/polyethylene glycol results in self-healing properties in addition to high elasticity (0.63 MPa, elongation at break 1200%). Since the material is stretchable, dynamic color change under strain was possible. A sample that was initially red turned blue as it stretched from 0 to 70%. The color change was quantified by changes in the reflection spectra. The peak in reflection shifted from 616 to 483 nm. This color change was attributed to a reduction in the lattice spacing leading to a blue shift of the reflection peak (Figure 7C) [103]. This color change has been modeled assuming affine deformation

$$\lambda ={{\left(1+\varepsilon_{zz}\right)}^{-1/2}\lambda }_{0}$$

where *ε_zz_* is normal elongational strain (i.e., uniaxial tensile strain along the long axis (*z*) of the filament), *λ*_0_ is the initial peak in the reflected wavelength, and *λ* is the reflected wavelength as the sample is stretched [97]. This model assumes a Poisson’s ratio of 0.5, i.e., the material was isotropic and incompressible. Experimentally, the Poisson’s ratio was estimated to be 0.49 in the lateral dimension and 0.57 in the vertical direction indicating anisotropic lateral shrinkage. Thus, deviation from the predicted wavelength is observed for non-affine deformation. Notably, the model provides a reasonable prediction of the wavelength as a function of strain during affine deformation, which corresponds to the visible color range [97]. The color change was reversible when the tensile force was removed. After releasing the stretch, the structural color returned to the initial state (red) indicating the initial lattice spacing was restored. The material showed self-healing properties; the mechanical properties were restored after cutting. The mechanochromic response was restored after 2 h of healing time [104]. Similar results have been achieved using other materials as the matrix. For example, vitrimers based on the polycondensation of polytetrahydrofuran (PTMG), glycerol (GLY), 2-hydroxyethyl disulfide (HEDS), and isophorone diisocyanate (IPDI) have also been considered as the matrix for the colloidal assemblies. The resulting materials are mechanochromic, transitioning from orange to green when stretched from 0 to 70% strain, with self-healing properties due to the vitrimer [108].

The mechanochromic sensitivity has been enhanced by patterning the particle assemblies. Specifically, templates containing arrangements of cuts of vertical double-crosses and horizontal lines with cuts of short vertical lines (Figure 7D) were prepared using stereolithography. The particle dispersion was cast on a glass plate with the template and another glass plate was placed on top. When removed from the template and glass plates, the resulting photonic material was structurally colored and demonstrated auxetic mechanical properties with a negative in-plane Poisson’s ratio due to the hierarchical cut design. The bulk photonic material turned from red to blue at 45% strain. Due to strain amplification in the patterned areas, enhanced strain sensitivity was achieved and tuned by adjusting the widths of the patterns. A maximum sensitivity of 60 nm/% was achieved. The resulting materials were used for visible structural health monitoring [105].

The advanced processing of colloidal dispersions has facilitated the design of sophisticated structurally colored materials. For example, colloidal dispersions (PS core and silica shell particles dispersed in ethanol) have been patterned on PDMS using masks to achieved colored patterns (e.g., heart, cat, etc.). The assembled particles were infiltrated with PDMS and a third layer of PDMS was added to achieve a three-layer sandwich PDMS/PS@SiO_2_/PDMS composite film. The patterns changed color when stretched and released, which are promising for anti-counterfeiting applications and smart labels [109]. Particle dispersions (silica particles in di(ethylene glycol) ethyl ether acrylate DEGEEA/polyethylene glycol mono-phenyl ester acrylate PEGPEA) were also patterned for responsive display applications [100].

Substrate selection can also enable functional applications. For example, colloidal particles can be self-assembled, infiltrated with polymer, and attached to textile substrates during polymerization to achieve textile-based sensors with mechanochromic properties. For example, the structural color of an orange-colored sensor changed gradually from orange to green when stretched from 0% to 20%. Quantitatively, the reflection peak continuously shifted from 604 to 558 nm as it was stretched from 0 to 51.6%. The color change was reversible (orange to green) when strained to 30% for at least 30,000 cycles [104]. Alternatively, colloidal particles can be self-assembled into cylindrical geometries to achieve structurally colored, responsive fibers. For example, monodisperse silica particles were dispersed with PDA particles and PEGPEA. Using a microfluidic device, the silica dispersion was simultaneously injected with PEGPEA saturated in hexadecane into a cross-junction to self-assemble colloidal fibers. As the jet flowed out from the device, it was continuously irradiated with UV to cure the fibers. The resulting fibers were structurally colored due to the rectangular particle array on the particle surface and responsive. For example, a blue shift was observed (from 629 nm to 499 nm) as the fiber was stretched longitudinally from 0 to 80% (Figure 7E). The color change from red to green (reflectance peak 625 nm to 543 nm) when strained from 0 to 40% was reversible for at least 10 cycles. The resulting fibers could be woven into a photonic fabric [106]. Another approach has been to coat colloidal particle assemblies on individual fibers. For example, iron oxide (Fe_3_O_4_ core, carbon-shell) particles in a PDMS matrix were coated on silicone fibers via dip coating. The fibers showed structural color due to the assembly of the particles. The color of the fiber changed (with a change in reflection spectra) when stretched to 50% strain [110]. Photonic fibers (silica/PEGPEA) have also been coated with a photopolymerizable ionic liquid fiber shell based on choline chloride and acrylic acid to achieve fibers that are both mechanochromic and conductive. The fibers could be incorporated into spandex and used to detect human motion [111].

Colloidal particles have also been formulated for additive manufacturing to achieve structurally colored, responsive 3D printed parts. Three-layer particles (polystyrene core and polymethacrylate interlayer/shell) were prepared by an emulsion polymerization of the core, followed by a polymerization of the interlayer, and then a polymerization of the shell. The volume of the fraction of the soft polymer shell was 53–54% to facilitate self-assembly into structurally colored materials and stable extrusion while preventing crosslinking. The refractive index contrast between the core and shell had to be as high as possible to maximize color intensity. The resulting particles were lyophilized, mixed with carbon black (0.03 wt.% to absorb diffusely scattered light), and extruded at 120 °C. The extruded strand was granulated and used for 3D printing using Fused Filament Fabrication. The 3D printed objects were structurally colored due to the Bragg diffraction of the colloidal crystal. Based on tensile testing, the yield strain was ~40%, and the tensile modulus was 1.7–1.8 MPa. Mechanochromic properties were observed upon stretching. For a sample that was initially red, a transition from red to green was observed upon increasing the strain from 0 to 30% (Figure 7F). More advanced 3D shapes (e.g., elf, house, gorilla, gecko, etc.) with structural color were also produced [98]. Alternatively, chemically crosslinked systems have also been formulated for 3D printing. For example, monodispersed poly(butyl acrylate) (PBA) spheres loaded with 2-ethylhexyl acrylate monomers (130–230 nm in diameter) were suspended with acrylamide and a photoinitiator and used for direct ink writing. Following curing, the particles were covalently linked, forming a continuous network; the maximum strain at break was ~2500%. The resulting free-standing materials showed structural color due to the assembly of the particles [112].

As an alternative to colloidal crystals, another approach to responsive, structural color has been periodic nanostructures obtained using top-down methods such as lithography or layered materials [4]. Structural color achieved from layers of materials with alternating refractive indexes can be calculated by simplified Bragg’s law when the incident angle is 90°

$$m\lambda =2(n_{1}d_{1}+n_{2}d_{2})$$

where *m* is the diffraction order, *λ* is the wavelength of reflected light, *n*_1_ and *n*_2_ are the refractive indexes of the alternating layers, and *d*_1_ and *d*_2_ are the layer thickness [4,113].

**Figure 8:**
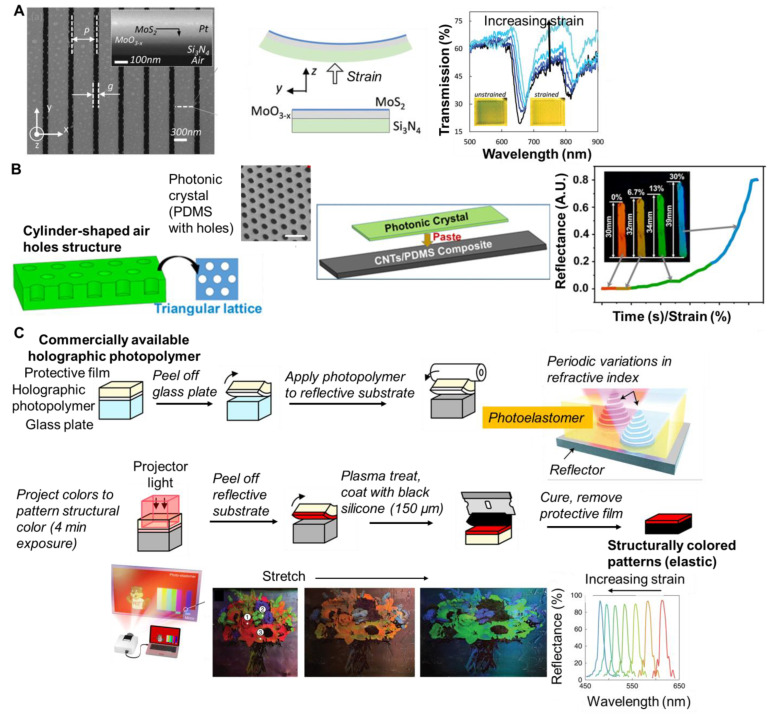
Overview of photonic metamaterials with mechanochromic properties. (**A**) Molybdenum disulfide, MoS_2_-based metamaterial from deposition of MoS_2_ (strain-sensitive refractive index) on a nanograting. Changes in transmission were observed upon applying strain. Arrow indicates spectra with increasing strain. Adapted with permission from [114]. (**B**) Photonic PDMS structure with periodic arrays of cylindrical holes prepared using a mold fabricated using nanolithography. The photonic material was layered on top of black PDMS for visual strain sensing. Adapted with permission from [88]. (**C**) Patterning of photonic nanostructures on commercially available elastomeric photopolymer was achieved using a standard light project and reflecting surface. The resulting structurally colored image was bonded to black silicone; complex patterns such as flowers have been achieved. Materials were mechanochromic showing a blue shift with applied strain. Adapted from [116] (CC-BY-SA-2.0).

Such nanostructured materials can be designed with responsive optical properties. For example, photonic metamaterials with mechanochromic properties have been investigated. Mechanochromic properties can be achieved using molybdenum disulfide, MoS_2_, since its refractive index is strain sensitive. Karvounis et al. deposited MoS_2_ on a Si_3_N_4_ nanograting-based metamaterial (gap width 100 nm, period 500 nm). Transmission changes up to 197% at 654 nm upon 2% of mechanical strain were observed (Figure 8A) and the mechanochromic response could be controlled by varying the speed of induced mechanical stress [114]. Nanogratings have also been 3D printed. For example, a mold with linear gratings with a period of 800 nm and line width of 90 nm were 3D printed with polylactic acid (PLA). PDMS was poured into the template (gravity filling). After demolding, PDMS with photonic structures were achieved. The as-prepared photonic film showed iridescent color (i.e., angle-dependent structural color). Mechanochromic properties were observed. The peak reflectance decreased from 635 nm to 504 nm (blue shift) when increasing from 0 to 100% strain parallel to the grating direction. Interestingly, the peak reflectance increased from 473 nm to 650 nm (red shift) when increasing the strain from 0 to 80% when applying strain perpendicular to the grating direction. The photonic material was clamped to carbon steel during a tensile stress and used as a colorimeter strain sensor with the color change quantified using chromaticity (CIE 1931) [115].

Alternatively, periodic nanostructures from PDMS have been fabricated using nanoimprinting to create a mold of periodic nanoscale cylinder nanostructures with a 300 nm diameter and 300 nm height and arranged as a non-close-packed triangular lattice with a period of 600 nm. The mold was filled with PDMS, and upon demolding, the resulting structure PDMS film contained non-close-packed cylinder-shaped air holes in a periodic nano-array, where the diameter of the holes was 300 nm and the period was 600 nm (Figure 8B). The resulting films showed structural color (initially red). As the photonic material was stretched, the lattice constant of the materials decreased, resulting in the color change from red to green to blue (when stretched to 29% strain). The color change was reversible over 2000 cycles (when stretched up to 50%). The photonic material was layered on top of a PDMS/carbon nanotube composite (black and conductive) to achieve a visual strain sensor. Due to the mechanochromic properties, the sensor could be used to visualize with good sensitivity at strains less than 30% (Figure 8B) [88].

The patterning of photonic nanostructures has also been achieved using a commercially available elastomeric photopolymer. Using a standard light project and reflecting surface, the image was recorded as periodic variations in refractive index that acted as distributed Bragg reflectors. After patterning, the photopolymer with the resulting structurally colored image was bonded to black silicone for mechanical tunability and to enhance the saturation of reflected colors. Complex patterns such as flowers have been achieved (Figure 8C). The resolution and image area was dictated by the size and resolution of the projected images; the minimum pixel size achieved was 10 µm. For an area with red structural color, the reflection spectrum shifted towards a lower wavelength (i.e., blue shift), as the applied strain increased. The color change due to mechanical strain was predicted based on a mechanical model and the approximation of the materials’ Poisson’s ratio as

$$\lambda_{max}=\lambda_{max,\;initial}{\left(1+\varepsilon_{L}\right)}^{-\nu }$$

where *ε_L_* is the applied uniaxial strain along the long axis of the sample, *λ_max_*_,_*_initial_* is the peak reflection wavelength at zero strain, and *ν* is the Poisson ratio (~0.5 for common elastomers). The color change was reversible for thousands of cycles and the color was stable at elevated temperature. The resulting materials were used for colorimetric mechanosensing. Hue (HSV color space) was used as the basis for quantitative colorimetric strain calibration and mapping [116]. To expand the capabilities in visual-based strain sensing, machine learning has been used to correlate strain to color using HSV descriptors from the digital image for mechanochromic photonic materials (silica particles in PEGDA) [117].

Nanostructured materials that facilitate responsive, structural color are promising mechanochromic systems. Such materials are promising in visual force/strain sensors. The range of detection, sensitivity, and stability for practical applications are currently being investigated [99]. Emerging applications such as materials that can hide and reveal images/messages and color shifting properties are of interest in anti-counterfeiting applications to identify authenticity and prevent forgery of products. Other applications have been recently reviewed elsewhere [89,99]. When used, their advantages include stable, tunable colors. However, scratches or cracks during use can cause scattering and reduce the quality. Additionally, photonic structures are often fragile with poor flexibility. Defects (microscopic or macroscopic) during preparation can hinder their practical application. Practically, their mechanical properties, durability, and stability need to be improved [89]. Achieving high throughput production for such materials remains a significant challenge [2,99].

## 7. Hydrogels

Hydrogels are used widely in mechanochromic material systems. For example, they can be used as matrix materials for mechanochromic dyes [25,39], liquid crystal droplets [54], and colloidal particle assemblies [112]. Interestingly, hydrogels alone can also form mechanochromic materials via self-assembly into layered structures (which can be considered a subset of photonic mechanochromic systems).

**Figure 9:**
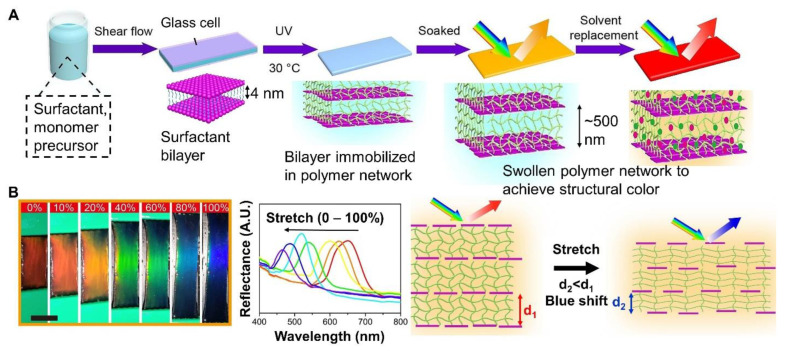
Overview of mechanochromic hydrogels. (**A**) Surfactant molecules self-assembled into two-dimensional bilayer lamellar structures and embedded in a cross-linked hydrogel matrix. When swollen in an appropriate solvent, reflective color was achieved. (**B**) Representative mechanochromic properties demonstrating visible color change from red to blue when stretched from 0 to 100% strain (confirmed with reflectance spectra) (1 cm scale bar). The blue shift was attributed to a decrease in lamellar spacing upon stretching. Arrows indicate structural color due to Bragg reflection. Adapted with permission from [118].

For example, mechanochromic hydrogels have been achieved by embedding bilayers of self-assembled surfactant in hydrogel matrices (Figure 9A). Specifically, N,N-dimethyl-1-dodecylamine N-oxide (C_12_DMAO), an amphoteric surfactant can self-assemble into 2D bilayer lamellar structures in water using n-hexanol as a co-surfactant. Following polymerization, and swelling in an appropriate solvent to achieve lamellar spacing on the order of the wavelength of visible light, structural color was achieved. The lamellar spacing was tuned by varying the crosslinker concentration and the ionic strength of the solvent used to swell the network. Thus, the initial color was changed from red to blue. Samples that were initially red changed to blue when uniaxially stretched [93].

In another example, mechanochromic hydrogels made with glycerol monolaurate bilayers, poly-(diacetone acrylamide-co-acrylamide) hydrogel, PEG 200, and ionic liquid (1-Ethyl-3-methylimidazolium bis(trifluoromethylsulfonyl)imide solvent turned from red to blue when stretched from 0 to 100% strain (confirmed with reflectance spectra). The blue shift was attributed to a decrease in lamellar spacing upon stretching (Figure 9B) [118].

Overall self-assembly of bilayers is a versatile biometric strategy to achieve responsive materials with structural colors. Responsive structural color can be combined with conductive properties for sensing applications. These bioinspired designs can mimic the unique properties of cephalopods [93,118], chameleons [96,109], and humans [54] due to their broadly tunable properties.

## 8. Hybrid Systems

**Figure 10:**
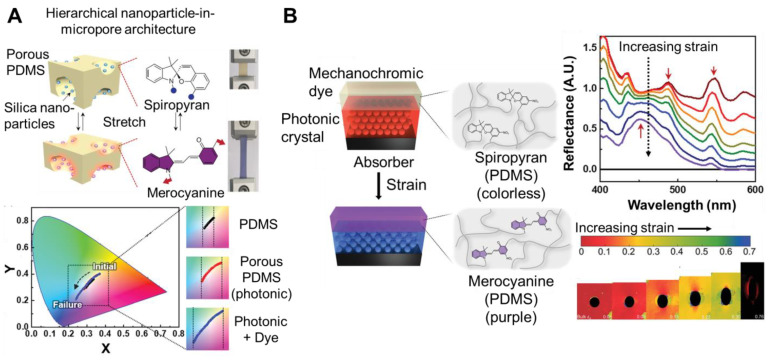
Overview of hybrid mechanochromic materials. (**A**) Porous PDMS-microparticle dye composites with hierarchical structure that turns from yellow to blue with strain due to spiropyran that shows an enhanced color transition region compared to dye in PDMS quantified using chromaticity. Adapted with permission from [120]. (**B**) Dual mode mechanoresponsive material that integrated photonic crystal (silica particles in PEG base layer) and absorptive dye (PDMS-spiropyran). Color change with strain due to the dye and photonic crystal was observed in the reflectance spectra (black arrow dotted arrow indicates spectra taken at increasing strain, red arrows highlight changes in the spectra). Using hyperspectral imaging, complex strain and stress distributions around defects were visualized. Adapted with permission from [119].

Building on these approaches to achieve responsive, structurally colored materials, additional hybrid approaches have combined the various classes of mechanochromic materials. For example, dyes combined with photonic structure color (e.g., [119]) or dyes combined with structurally colored liquid crystal elastomers [6] have been explored to enhance the functional mechanochromic properties such as dynamic color range (i.e., range of colors observed upon strain). For example, spiropyran has been incorporated in porous PDMS with a hierarchical structure (Figure 10A) achieved by mixing spiropyran, silica nanoparticles, and PDMS with hydrophilic cosolvents (water and ethanol). Followed by the evaporation of the solvents during the hydrosilylation curing process, spherical pores were formed within the composites as a result of phase separation between the hydrophobic PDMS matrix and hydrophilic solvents. The films were then freeze dried to eliminate residual solvents. The resulting porous composite films were light yellow and turned to blue at 200% tensile strain due to the mechanochemical ring-opening of spiropyran to merocyanine. Based on luminescence spectra, the color change was reversible upon releasing the strain. The color returned to the original color after 35 s for a strain of 175%. The recovery time increased with increasing tensile strain. An analysis of color change using chromaticity (CIE 1931) indicated that the spiropyran in the porous, hierarchical structure exhibited a larger color range than spiropyran in PDMS or spiropyran in porous PDMS (Figure 10A). This effect was attributed to effective stress concentration in the hierarchical structure at the silica nanoparticle–micropore interface as well as improved stretchability [120]. In a similar approach, mechanochromic properties have also been achieved using dye/clay/polymer composites. A photoluminescence change from red to blue was observed using cyanine dye with smectite (clay) in gelatin. The mechanochromism was explained by the structural distortion of the dye clusters confined within the clay [76].

In another approach, silica nanoparticles in PEG elastomer (poly(ethylene glycol) phenyl ether acrylate, PEGPEA) was used as a base layer (on top of PDMS with carbon black). A layer of PDMS with covalently linked spiropyran was layered on top to increase the dynamic range (Figure 10B). The dye layer was initially colorless. When enough strain was applied, the dye turned purple due to the transition of spiropyran to merocyanine in addition to the blue shift due to the strain of the photonic crystal. These changes in strain were visualized using hyperspectral imaging and used to map complex strain distributions around defects (Figure 10B) [119]. A similar approach was used with a photonic film (PDMS matrix with rhodamine B cast atop a photonic array of silica nanoparticles). The composite silica nanoparticle/PDMS/rhodamine B film turned from transparent, amber to opaque, pink at 100% strain when viewed macroscopically under ambient conditions. The color change was attributed to the dye; the change in transparency was attributed to the void spaces in the photonic layer. The visible changes were reversible over 1100 cycles [121].

Overall, hybrid systems are an emerging approach to achieve dynamic structural color with enhanced functional properties. The responsive properties of the composite materials have been inspired by biological systems such as cephalopods and chameleons with a wide range of potential applications. However, with the increased intricacies of materials processing the trade-off between increased performance and complexity should be carefully considered [122].

## 9. Comparison of Classes of Stretchable Mechanochromic Materials

**Figure 11:**
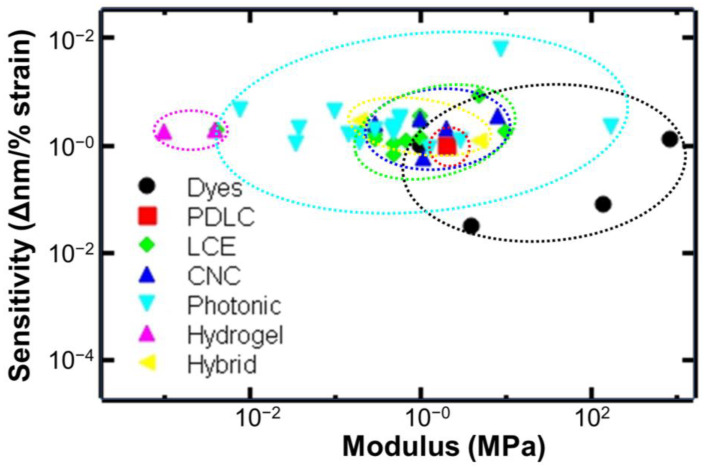
Comparison of mechanochromic sensitivity (Δnm/%strain) as a function of elastic modulus (MPa) for various classes of mechanochromic materials Each class of material is grouped by the dotted lines to guide the eye [1,6,17,32,40,48,49,55,62,64,66,67,68,73,75,78,80,81,83,93,95,96,97,100,102,103,104,105,107,108,110,111,112,113,115,118,119,124,125,126,127].

Various classes of mechanochromic materials that change color with stretch have been achieved, including the following: dyes, polymer dispersed liquid crystals, liquid crystal elastomers, cellulose nanocrystals, photonic liquid crystals, hydrogels, and hybrid systems (combinations of other classes). A figure of merit that can be used to compare the color change across different classes of mechanochromic materials has been the mechanochromic sensitivity, i.e., the amount of color change per tensile strain applied reported in Δnm/% strain [123]. The higher the sensitivity, the lower the amount of strain required to achieve an optical response. Data for mechanochromic sensitivity and elastic modulus for various classes of mechanochromic materials have been compiled (available in the Supporting Information) A comparison of the functional performance of the different classes of materials in terms of mechanochromic sensitivity in units of (Δnm/%strain) as a function of the elastic modulus of the composite materials is provided in Figure 11. 

The hydrogel systems had the lowest modulus. Dye systems had higher moduli and lower mechanochromic sensitivity. The most data were available for photonic and liquid crystal elastomer systems, which have been tuned over a wide range of elastic moduli. Large shifts in visible color resulted in wavelength differences of ~200 nm [46]; therefore, the mechanochromic sensitivities were comparable ~1–2 nm/% strain. The highest sensitivity 60 nm/% strain was reported by patterning colloidal photonic particle assemblies [105].

Mechanochromic sensitivity is a useful metric for comparing classes of materials. However, other factors may also affect the functional performance of the mechanochromic material. For example, the range of colors that can be obtained (dynamic color range) may be of practical importance. Dye systems typically can achieve a single-color transition, e.g., yellow to purple. Multiple dyes can then be combined into a single system to achieve multiple color switches [33]. Liquid crystals elastomers can be formulated to undergo changes in transmission (opaque to transparent) [60] or demonstrate structural color in the visible range. Structurally colored systems (liquid crystal elastomer, polymer dispersed liquid crystals, photonic, and hydrogels) can be designed to reflect all colors in the visible spectrum (violet to red). Hybrid systems combining dyes and photonic structures can increase the dynamic color range [120].

The reversibility of the color change may also be an important consideration for practical applications. For dyes, the color change (due to bond cleavage) is typically reversible with external stimuli such as heat or light over minutes to hours. The reversibility of the color change of liquid crystal elastomer systems alone can also be relatively slow. Commonly, liquid crystal elastomers are layered with elastomeric films to increase the rate of color change reversal [61]. For polymer dispersed liquid crystal systems and photonic systems, the color change is considered instantaneous (~milliseconds), but is rarely fully characterized.

To predict the mechanochromic behavior, models can be useful for relating the change in wavelength to the change in strain. For structurally colored systems, such as liquid crystal elastomers and photonic systems, mechanical models have been used to relate the change in strain to the change in wavelength with reasonable agreement with experimental results in the visible range. For dyes, molecular-based modeling approaches have been used to predict forces required for bond cleavage. However, reconciling the theoretically calculated forces on single molecules with the macroscopically measured stresses required for color changes is an ongoing area of investigation [40]. For cellulose nanocrystals, models of structural color are typically based on mesogen orientation quantified by the order parameter (measured by X-ray scattering). To understand the effect of anisotropic deswelling or stretch, the compression matrix formalism has been used [85,86]. Such an approach may also be appropriate for polymer dispersed liquid crystals, but to date, has not been applied. Mechanical models (e.g., Maxwell model) have also been used for mechanochromic liquid crystal/polymer systems [128].

An overall summary comparing the functional properties affecting the practical performance and potential applications of mechanochromic materials is provided in Table 1. Photonic systems achieve a high sensitivity (i.e., large Δnm/% strain), large dynamic color range, and rapid reversibility (e.g., [105]). Further, their mechanochromic behavior can be predicted using a simple mechanical model [103,113]. Systems with a wide range of mechanical properties (elastic moduli) have been achieved (Figure 11). The addition of dyes to photonic systems can further improve the dynamic range (range of strain/force over which there is an optical change) [120]. For applications in which irreversible color change is desirable, dye-based systems [32] or liquid crystal elastomer systems [61] can be formulated.

## 10. Conclusions and Outlook

### 10.1. Conclusions

The mechanochromic sensitivity (Δnm/% strain) is a useful figure of merit for comparing different classes of mechanochromic materials. Dye systems had higher moduli and lower mechanochromic sensitivity compared to other classes of mechanochromic materials. Photonic and liquid crystal elastomer systems have been tuned over a wide range of elastic moduli with large shifts in visible color of ~200 nm resulting in mechanochromic sensitivities of ~1–2 nm/%strain. The highest sensitivity 60 nm/% strain was achieved by patterning colloidal photonic particle assemblies. Other important functional properties of mechanochromic systems include dynamic color range (e.g., transparent to opaque, or red to blue) as well as the rate of reversibility (e.g., minutes to hours for dyes or liquid crystal elastomers or instantaneous for photonic). The comparison of functional properties provided in this review is intended to facilitate materials selection for a wide variety of applications. 

### 10.2. Outlook

Based on this work, it is possible to quantitatively compare the performance of different classes of mechanochromic materials using the mechanochromic sensitivity (Δnm/% sensitivity). The standardized reporting of mechanical properties (e.g., elastic modulus and type of strain) as well as color measurements (e.g., reflected peak position) would increase the amount of data available for comparison. For applications in visual detection, standardized methods in imaging (e.g., lighting, camera setting, etc.) as well as a quantitative analysis of the digital color (e.g., RGB values) would be especially valuable. Techniques in computer vision may be especially useful in leveraging the spatiotemporal information that mechanochromic materials can provide.

Overall, mechanochromic materials are versatile with a wide range of potential applications. Dynamically tunable structural color may enable novel functionalities such as color printing animations, security encryption, and sensing applications [2]. Mechanochromic materials provide optical changes without the need for external monitoring devices and 3D printing enables the fabrication of sensors of complex geometries [3]. Thus, stress visualization as the color changes can be correlated with stress intensity. Such materials may be applied as mechanical sensors in physics, health care, and engineering. For example, when incorporated into fibers, the color has been used as a sensor to monitor the applied pressure on wound dressings [4]. Mechanochromic materials have also been incorporated with liquid metals in elastomers to achieve mechanochromic stretchable electronics for failure sensing [3]. Applications have included crack detection for infrastructure [4]. Mechanochromic materials incorporated into ionic gels mimicked human skin sensing mechanical stimuli (touch) and underwent color change (bruising) when subjected to heavy loads [3]. Due the responsive optical properties, application in smart windows with fast response times [4,5] have also been proposed. Mechanochromic materials have been proposed for used as optical filters in ultracompact MEMS (Micro-Electro-Mechanical systems) based spectrophotometers [2].

While many promising applications have been demonstrated, manufacturing uniform color on a large scale remains a challenge. Image analysis to convert color change to quantitative readouts remains a significant challenge [4]. Thus, standardized characterization methods are needed to translate materials to practical application. The ability to tune the sensitivity of the mechanochromic response, e.g., the onset stress/strain of color activation and the range of activation energy, is also of practical interest [3]. The sustainability of mechanochromic materials is also an important consideration. For example, dynamic covalent chemistries may also enable recycling. The use of materials from renewable sources instead of petrochemicals is also an important consideration (e.g., cellulose nanocrystals and biobased monomers) [59,87].

## Figures and Tables

**Table 1:** Comparison of the functional properties affecting the performance/applications of mechanochromic materials.

Class of Stretchable Mechanochromic Materials	Sensitivity	Dynamic Color Range	Color Change Reversibility	Model
Dyes	+	+	+	+
PDLC	++	+++	+++	-
LCE	++	+/+++	+/+++	+++
Cellulose Nanocrystal	++	+++	+++	+++
Photonic/hydrogel	+++	+++	+++	+++

+++ indicates high performance relative to other material classes, ++ indicates performance comparable to other material classes, + indicates low performance relative to other material classes, - indicates not available/indicates performance depends on formulation.

## Data Availability

The original contributions presented in the study are included in the article/Supplementary Materials. Further inquiries can be directed to the corresponding author.

## Supplementary Materials

The following supporting information can be downloaded at: https://www.mdpi.com/article/10.3390/ma17163980/s1, Table S1: Compiled mechanochromic sensitivity and elastic modulus data.

## Abbreviation

3D	three-dimensional
5CB	4-Cyano-4-pentylbipheny
AM	acrylamide
BDB	dithiol-functionalized boronic ester 2,2′-(1,4-phenylene)-bis[4-mercaptan-1,3,2-dioxaborolane]
BBS	bis(benzoxazolyl)stilbene
BHT	butylated hydroxy toluene
c	concentration
C12DMAO	N,N-dimethyl-1-dodecylamine N-oxide
CIE	International Commission on Illumination
CLC	cholesteric liquid crystal
CNC	cellulose nanocrystal
CSP	core shell particle
d	particle diameter
D	lattice constant
DABBF	diarylbibenzofuranone
DABBT	diarylbibenzothiophenonyl
DCM	dichloromethane
DEGEEA	di(ethylene glycol) ethyl ether acrylate
E	Young’s modulus
E7	mixture of 4-Cyano-4′-pentylbiphenyl, 4′-Heptyl-4-biphenylcarbonitrile, 4′-Octyloxy-4-biphenylcarbonitrile, and 4-[4-(4-pentylphenyl)phenyl]benzonitrile
EDDET	2,2′-(ethylenedioxy) diethanethiol
Glu	glucose
GLY	glycerol
HEA	2-hydroxyethyl acrylate
HEDS	2-hydroxyethyl disulfide
HSV	hue, saturation, and value
HTP	helical twisting power
Igracure 651	2,2-dimethoxy-1,2-diphenylethan-1-one
IPDI	isophorone diisocyanate
LC756	2,5-Bis-O-[4-[[4-[[[4-(acryloyloxy)butoxy]carbonyl]oxy]benzoyl]oxy]benzoyl]-1,4:3,6-dianhydro-D-glucitol
LCE	liquid crystal elastomers
LDPE	low density polyethylene
m	order of diffraction
MA	methyl acrylate
MEMS	Micro-Electro-Mechanical systems
n	index of refraction
NIPAM	N-Isopropylacrylamide
OPVs	oligo(p-phenylenevinylene)s
p	helical pitch
PAM	poly(acrylamide)
PAMPS	poly(2-acrylamido-2-methyl-1-propanesulfonic acid)
PBT	poly(butylene terephthalate)
PCL	polycaprolactone
PDA	polydopamine particles
PDLC	polymer dispersed liquid crystals
PDMS	Polydimethylsiloxane
PEGDMA	poly(ethylene glycol) dimethacrylate
PEGPEA	poly(ethylene glycol) phenyl ether acrylate,
PET	polyethylene terephthalate
PETMP	pentaerythritol tetrakis (3-mercaptopropionate)
PMMA	poly(methyl methacrylate)
PLA	polylactic acid
(PS)	polystyrene
PTMG	polytetrahydrofuran
PVA	polyvinyl alcohol
R5011	(13bR)-5,6-Dihydro-5-(trans-4-propylcyclohexyl)-4H-dinaphtho[2,1-f:1′,2′-h][1,5]dioxonin
(RM257)	1,4-Bis[4-(3-acryloyloxypropyloxy)benzoyloxy]-2-methylbenzene
RGB	red, green, blue
RhB	rhodamine B
S811	S-(+)-2-Octyl 4-(4-hexyloxybenzoyloxy)benzoate
SEM	scanning electron microscopy
SP	spiropyran
TEA	triethylamine
TPU	thermoplastic polyurethane
UV	ultraviolet
θ	angle of incident light
λ	wavelength of reflected light
ν	Poisson’s ratio
ϕ	volume fraction
