# Supplementary material for: Fundamental Aspects of Stretchable Mechanochromic Materials: Fabrication and Characterization
Source: Materials (Basel). 2024 Aug 10;17(16):3980. doi: 10.3390/ma17163980 (PMC11355797; doi:10.3390/ma17163980)
Supplement: Supplementary file 1 [file materials-17-03980-s001.zip › materials-3088995-supplementary.pdf]

Supporting Information

Fundamental Aspects of Stretchable Mechanochromic Materials: Fabrication and Characterization

Christina Tang

Chemical and Life Science Engineering, Virginia Commonwealth University, Richmond, VA, 23294-3028
*Correspondence: ctang2@vcu.edu

Table S1. Compiled mechanochromic sensitivity and elastic modulus data

Material System	Elastic modulus (MPa)	Mechanochromic Sensitivity (Δnm/% strain)	Reference
Dye 1	140	0.075	[1]
Dye 2	1	1	[2]
Dye 3	4	0.03	[3]
Dye 4	820	1.25	[4]
PDLC 1	2	1	[5]
LCE 1	0.5	0.65	[6]
LCE 2	10	1.8	[7]
LCE 3	0.7	1.23	[8]
LCE 4	0.3	1.59	[9]
LCE 5	1	2.73	[10]
LCE 6	0.5	1.067	[11]
LCE 7	1	3.35	[12]
LCE 8	1	1.33	[13]
LCE 9	5	8.2	[14]
LCE 10	0.3	1.26	[15]
LCE 11	0.0043	1.95	[16]
Cellulose nanocrystal 1	1	2.98	[17]
Cellulose nanocrystal 2	0.3	2.5	[18]
Cellulose nanocrystal 3	8	3.37	[19]
Cellulose nanocrystal 4	0.4-1.1	0.59	[20]
Cellulose nanocrystal 5	2	2.01	[21]
Photonic -1	1.3	0.88	[22]
Photonic -2	103	2.98	[23]
Photonic -3	0.1	4.15	[24]
Photonic -4	0.5	1.71	[25]
Photonic -5	0.588	3.16	[26]
Photonic -6	0.038	1.98	[27]
Photonic -7	10	1.1	[28]
Photonic -8	170	1.31-2.21	[29]
Photonic -9	0.5	2.24	[30]
Photonic -10	0.187	1.9	[31]
Photonic -11	0.150	1.51	[32]
Photonic -12	0.035	1.06	[33]
Photonic -13	0.2	1.6	[34]
Photonic -14	8.93	60	[35]
Photonic -15	3	1.2	[36]
Photonic -16	0.47	1.85	[28]
Photonic 17	0.0082	4.59	[37]
Hydrogel 1	0.001	1.78	[38]
Hydrogel 2	0.004	1.95	[39]
Hybrid 1	0.2	2.75	[40]
Hybrid 2	5	1.2	[41]

References

1. Jang, H.G.; Jo, J.Y.; Park, H.; Jung, Y.C.; Choi, Y.S.; Jung, S.; Lee, D.C.; Kim, J. Mechano-Responsive Spiropyran Microbeads: A Facile Fabrication Strategy for Self-Reporting Materials. *Adv Mater Technol* **2023**, *8*, doi:10.1002/admt.202200566.

2. Gossweiler, G.R.; Hewage, G.B.; Soriano, G.; Wang, Q.; Welshofer, G.W.; Zhao, X.; Craig, S.L. Mechanochemical Activation of Covalent Bonds in Polymers with Full and Repeatable Macroscopic Shape Recovery. *ACS Macro Lett* **2014**, *3*, 216–219, doi:10.1021/mz500031q.

3. Raisch, M.; Maftuhin, W.; Walter, M.; Sommer, M. A Mechanochromic Donor-Acceptor Torsional Spring. *Nat Commun* **2021**, *12*, doi:10.1038/s41467-021-24501-1.

4. Lott, J.; Weder, C. Luminescent Mechanochromic Sensors Based on Poly(Vinylidene Fluoride) and Excimer-Forming *p* -Phenylene Vinylene Dyes. *Macromol Chem Phys* **2010**, *211*, 28–34, doi:10.1002/macp.200900476.

5. Balenko, N. V.; Shibaev, V.P.; Bobrovsky, A.Y. Mechano-Optical Response of Novel Polymer Composites Based on Elastic Polyurethane Matrix Filled with Low-Molar-Mass Cholesteric Droplets. *Macromol Mater Eng* **2021**, *306*, 1–10, doi:10.1002/mame.202100262.

6. Kizhakidathazhath, R.; Geng, Y.; Jampani, V.S.R.; Charni, C.; Sharma, A.; Lagerwall, J.P.F. Facile Anisotropic Deswelling Method for Realizing Large-Area Cholesteric Liquid Crystal Elastomers with Uniform Structural Color and Broad-Range Mechanochromic Response. *Adv Funct Mater* **2020**, *30*, doi:10.1002/adfm.201909537.

7. Sun, C.; Zhang, S.; Ren, Y.X.; Zhang, J.; Shen, J.; Qin, S.; Hu, W.; Zhu, S.; Yang, H.; Yang, D. Force-Induced Synergetic Pigmentary and Structural Color Change of Liquid Crystalline Elastomer with Nanoparticle-Enhanced Mechanosensitivity. *Advanced Science* **2022**, *9*, doi:10.1002/advs.202205325.

8. Geng, Y.; Lagerwall, J.P.F. Multiresponsive Cylindrically Symmetric Cholesteric Liquid Crystal Elastomer Fibers Templated by Tubular Confinement. *Advanced Science* **2023**, *10*, doi:10.1002/advs.202301414.

9. Ma, J.; Yang, Y.; Zhang, X.; Wang, L.; Feng, W. Mechanochromic, Shape-Programmable and Self-Healable Liquid Crystal Elastomers Enabled by Dynamic Covalent Ester Bonds. *Angewandte Chemie - International Edition* **2022**, *61*, e202116219, doi:10.1002/anie.201911468.

10. Ku, K.; Hisano, K.; Kimura, S.; Shigeyama, T.; Akamatsu, N.; Shishido, A.; Tsutsumi, O. Environmentally Stable Chiral-nematic Liquid-crystal Elastomers with Mechano-optical Properties. *Applied Sciences (Switzerland)* **2021**, *11*, doi:10.3390/app11115037.

11. Geng, Y.; Kizhakidathazhath, R.; Lagerwall, J.P.F. Robust Cholesteric Liquid Crystal Elastomer Fibres for Mechanochromic Textiles. *Nat Mater* **2022**, *21*, 1441–1447, doi:10.1038/s41563-022-01355-6.

12. Zhang, P.; Shi, X.; Schenning, A.P.H.J.; Zhou, G.; de Haan, L.T. A Patterned Mechanochromic Photonic Polymer for Reversible Image Reveal. *Adv Mater Interfaces* **2020**, *7*, doi:10.1002/admi.201901878.

13. Choi, J.; Choi, Y.; Lee, J.H.; Kim, M.C.; Park, S.; Hyun, K.; Lee, K.M.; Yoon, T.H.; Ahn, S. kyun Direct-Ink-Written Cholesteric Liquid Crystal Elastomer with Programmable Mechanochromic Response. *Adv Funct Mater* **2024**, *34*, doi:10.1002/adfm.202310658.

14. Park, H.; Lee, H.J.; Ahn, H.; Han, W.C.; Yun, H.S.; Choi, Y.S.; Kim, D.S.; Yoon, D.K. Mechanochromic Palettes of Cholesteric Liquid Crystal Elastomers for Visual Signaling. *Adv Opt Mater* **2024**, doi:10.1002/adom.202400266.

15. Ma, J.; Yang, Y.; Zhang, X.; Xue, P.; Valenzuela, C.; Liu, Y.; Wang, L.; Feng, W. Mechanochromic and Ionic Conductive Cholesteric Liquid Crystal Elastomers for Biomechanical Monitoring and Human-Machine Interaction. *Mater Horiz* **2023**, *11*, 217–226, doi:10.1039/d3mh01386c.

16. Hussain, S.; Park, S.Y. Photonic Cholesteric Liquid-Crystal Elastomers with Reprogrammable Helical Pitch and Handedness. *ACS Appl Mater Interfaces* **2021**, *13*, 59275–59287, doi:10.1021/acsami.1c18697.

17. Zhang, Z.L.; Dong, X.; Zhao, Y.Y.; Song, F.; Wang, X.L.; Wang, Y.Z. Bioinspired Optical Flexible Cellulose Nanocrystal Films with Strain-Adaptive Structural Coloration. *Biomacromolecules* **2022**, *23*, 4110–4117, doi:10.1021/acs.biomac.2c00491.

18. Meng, Q.; Zhang, M.; Tang, R.; Jin, W.; Zhang, J.; Lan, Z.; Shi, S.; Shen, X.; Sun, Q. Stretchable Triboelectric Nanogenerator with Exteroception-Visualized Multifunctionality. *J Mater Chem A Mater* **2022**, *10*, 4300–4305, doi:10.1039/d1ta09825j.

19. Ge, W.; Zhang, F.; Wang, D.; Wei, Q.; Li, Q.; Feng, Z.; Feng, S.; Xue, X.; Qing, G.; Liu, Y. Highly Tough, Stretchable, and Solvent-Resistant Cellulose Nanocrystal Photonic Films for Mechanochromism and Actuator Properties. *Small* **2022**, *18*, doi:10.1002/smll.202107105.

20. Boott, C.E.; Tran, A.; Hamad, W.Y.; MacLachlan, M.J. Cellulose Nanocrystal Elastomers with Reversible Visible Color. *Angewandte Chemie - International Edition* **2020**, *59*, 226–231, doi:10.1002/anie.201911468.

21. Li, X.; Yang, Y.; Valenzuela, C.; Zhang, X.; Xue, P.; Liu, Y.; Liu, C.; Wang, L. Mechanochromic and Conductive Chiral Nematic Nanostructured Film for Bioinspired Ionic Skins. *ACS Nano* **2023**, *17*, 12829–12841, doi:10.1021/acsnano.3c04199.

22. Zhao, K.; Cao, X.; Alsaid, Y.; Cheng, J.; Wang, Y.; Zhao, Y.; He, X.; Zhang, S.; Niu, W. Interactively Mechanochromic Electronic Textile Sensor with Rapid and Durable Electrical/Optical Response for Visualized Stretchable Electronics. *Chemical Engineering Journal* **2021**, *426*, doi:10.1016/j.cej.2021.130870.

23. Lee, G.H.; Han, S.H.; Kim, J. Bin; Kim, J.H.; Lee, J.M.; Kim, S.H. Colloidal Photonic Inks for Mechanochromic Films and Patterns with Structural Colors of High Saturation. *Chemistry of Materials* **2019**, *31*, 8154–8162, doi:10.1021/acs.chemmater.9b02938.

24. Chen, J.; Xu, L.; Yang, M.; Chen, X.; Chen, X.; Hong, W. Highly Stretchable Photonic Crystal Hydrogels for a Sensitive Mechanochromic Sensor and Direct Ink Writing. *Chemistry of Materials* **2019**, *31*, 8918–8926, doi:10.1021/acs.chemmater.9b02961.

25. Miwa, E.; Watanabe, K.; Asai, F.; Seki, T.; Urayama, K.; Odent, J.; Raquez, J.M.; Takeoka, Y. Composite Elastomer Exhibiting a Stress-Dependent Color Change and High Toughness Prepared

by Self-Assembly of Silica Particles in a Polymer Network. *ACS Appl Polym Mater* **2020**, *2*, 4078–4089, doi:10.1021/acsapm.0c00703.

26. Yang, D.; Ye, S.; Ge, J. From Metastable Colloidal Crystalline Arrays to Fast Responsive Mechanochromic Photonic Gels: An Organic Gel for Deformation-Based Display Panels. *Adv Funct Mater* **2014**, *24*, 3197–3205, doi:10.1002/adfm.201303555.

27. Li, M.; Tan, H.; Jia, L.; Zhong, R.; Peng, B.; Zhou, J.; Xu, J.; Xiong, B.; Zhang, L.; Zhu, J. Supramolecular Photonic Elastomers with Brilliant Structural Colors and Broad-Spectrum Responsiveness. *Adv Funct Mater* **2020**, *30*, doi:10.1002/adfm.202000008.

28. Lee, G.H.; Choi, T.M.; Kim, B.; Han, S.H.; Lee, J.M.; Kim, S.H. Chameleon-Inspired Mechanochromic Photonic Films Composed of Non-Close-Packed Colloidal Arrays. *ACS Nano* **2017**, *11*, 11350–11357, doi:10.1021/acsnano.7b05885.

29. Zhang, R.; Yang, Z.; Zheng, X.; Zhang, Y.; Wang, Q. Large-Strain and Full-Color Change Photonic Crystal Films Used as Mechanochromic Strain Sensors. *Journal of Materials Science: Materials in Electronics* **2021**, *32*, 15586–15593, doi:10.1007/s10854-021-06107-x.

30. Miller, B.H.; Liu, H.; Kolle, M. Scalable Optical Manufacture of Dynamic Structural Colour in Stretchable Materials. *Nat Mater* **2022**, doi:10.1038/s41563-022-01318-x.

31. Zhao, M.; Ren, P.; Lyu, Q.; Chen, X.; Wang, H.; Li, M.; Zhang, L.; Zhu, J. Adhesive Photonic-Ionic Skins for Visualizing Wearable Strain Distributions. *Chemical Engineering Journal* **2023**, *470*, doi:10.1016/j.cej.2023.143937.

32. Zhao, R.; He, Y.; He, Y.; Li, Z.; Chen, M.; Zhou, N.; Tao, G.; Hou, C. Dual-Mode Fiber Strain Sensor Based on Mechanochromic Photonic Crystal and Transparent Conductive Elastomer for Human Motion Detection. *ACS Appl Mater Interfaces* **2023**, *15*, 16063–16071, doi:10.1021/acsami.3c00419.

33. Zhang, H.; Chen, H.; Lee, J.H.; Kim, E.; Chan, K.Y.; Venkatesan, H.; Shen, X.; Yang, J.; Kim, J.K. Mechanochromic Optical/Electrical Skin for Ultrasensitive Dual-Signal Sensing. *ACS Nano* **2023**, *17*, 5921–5934, doi:10.1021/acsnano.3c00015.

34. Wei, B.; Hu, Y.; Yang, D.; Huang, S. Sensitive Stretchable and Pressable Mechanochromic Photonic Crystals for Dynamic Displays and Visual Sensing. *Advanced Sensor Research* **2023**, *2*, doi:10.1002/adsr.202200078.

35. Lee, H.Y.; Gu, M.; Hwang, J.; Hwang, H.; Kim, Y.S.; Lee, S.Y.; Kim, S.H. Auxetic Photonic Patterns with Ultrasensitive Mechanochromism. *Advanced Science* **2024**, *11*, doi:10.1002/advs.202304022.

36. Guan, Y.; Li, H.; Zhang, S.; Niu, W. Mechanochromic Photonic Vitrimer Thermal Management Device Based on Dynamic Covalent Bond. *Adv Funct Mater* **2023**, *33*, doi:10.1002/adfm.202215055.

37. Yang, W.; Yamamoto, S.; Sueyoshi, K.; Inadomi, T.; Kato, R.; Miyamoto, N. Perovskite Nanosheet Hydrogels with Mechanochromic Structural Color. *Angewandte Chemie* **2021**, *133*, 8547–8552, doi:10.1002/ange.202015982.

38. Niu, W.; He, X.; Wang, Y.; Cao, X.; Cheng, J.; Yao, B.; Zhao, Y.; Wu, S.; Ju, B.; Zhang, S. Cephalopod-Inspired Chromotropic Ionic Skin with Rapid Visual Sensing Capabilities to Multiple Stimuli. *ACS Nano* **2021**, *15*, 3509–3521, doi:10.1021/acsnano.1c00181.

39. Wang, Y.; Zhang, H.; Zhang, S.; Niu, W. Squid-Inspired Photonic-Ionic Skin with Anti-Freezing, Drying-Tolerance, and Antibacterial Abilities for Wirelessly Interactive Multi-Sensing. *Chemical Engineering Journal* **2023**, *462*, doi:10.1016/j.cej.2023.142290.

40. Clough, J.M.; van der Gucht, J.; Kodger, T.E.; Sprakel, J. Cephalopod-Inspired High Dynamic Range Mechano-Imaging in Polymeric Materials. *Adv Funct Mater* **2020**, *30*, doi:10.1002/adfm.202002716.

41. Zhang, S.; Sun, C.; Zhang, J.; Qin, S.; Liu, J.; Ren, Y.; Zhang, L.; Hu, W.; Yang, H.; Yang, D. Reversible Information Storage Based on Rhodamine Derivative in Mechanochromic Cholesteric Liquid Crystalline Elastomer. *Adv Funct Mater* **2023**, *33*, doi:10.1002/adfm.202305364.
